# Designing an ecofriendly catalyst for a sustainable use of water resources

**DOI:** 10.1093/nsr/nwaf447

**Published:** 2025-10-20

**Authors:** Dehui Qiu, Xiaobo Zhang, Fan Tian, Yuan Liu, Fangni He, Xinrong Yan, Shijiong Wei, Jean-Louis Mergny, David Monchaud, Shujuan Zhang, Huangxian Ju, Jun Zhou

**Affiliations:** State Key Laboratory of Analytical Chemistry for Life Science, School of Chemistry & Chemical Engineering, Nanjing University, Nanjing 210023, China; State Key Laboratory of Analytical Chemistry for Life Science, School of Chemistry & Chemical Engineering, Nanjing University, Nanjing 210023, China; Key Laboratory of Green Chemical Process of Ministry of Education, School of Chemistry and Environmental Engineering, Wuhan Institute of Technology, Wuhan 430205, China; State Key Laboratory of Analytical Chemistry for Life Science, School of Chemistry & Chemical Engineering, Nanjing University, Nanjing 210023, China; State Key Laboratory of Analytical Chemistry for Life Science, School of Chemistry & Chemical Engineering, Nanjing University, Nanjing 210023, China; State Key Laboratory of Analytical Chemistry for Life Science, School of Chemistry & Chemical Engineering, Nanjing University, Nanjing 210023, China; State Key Laboratory of Analytical Chemistry for Life Science, School of Chemistry & Chemical Engineering, Nanjing University, Nanjing 210023, China; State Key Laboratory of Analytical Chemistry for Life Science, School of Chemistry & Chemical Engineering, Nanjing University, Nanjing 210023, China; Laboratoire d’Optique et Biosciences (LOB), Ecole Polytechnique, CNRS, INSERM, Institut Polytechnique de Paris, Palaiseau 91120, France; Institut de Chimie Moléculaire de l’Université de Bourgogne (ICMUB), CNRS UMR6302, Université Bourgogne Europe (UBE), Dijon 21078, France; State Key Laboratory of Pollution Control and Resource Reuse, School of the Environment, Nanjing University, Nanjing 210023, China; State Key Laboratory of Analytical Chemistry for Life Science, School of Chemistry & Chemical Engineering, Nanjing University, Nanjing 210023, China; State Key Laboratory of Analytical Chemistry for Life Science, School of Chemistry & Chemical Engineering, Nanjing University, Nanjing 210023, China

**Keywords:** artificial enzyme, chimeric peptide DNAzyme, catalytic mechanism, saving water, wastewater treatment

## Abstract

The printing and dyeing industry is one of the most polluting (∼20% of global clean water pollution), water-consuming and energy-wasting sectors in the manufacturing field, highlighting the need to find green catalysts to improve its sustainability. Herein, a novel artificial green catalyst was developed, known as a bifunctional chimeric peptide DNAzyme (bi-CPDzyme), comprising peptide, DNA and hemin moieties. This catalyst displays both catalase (CAT) and peroxidase (POD) activities. The turnover number (*k*_cat_) of the optimized bi-CPDzyme prototype (G-quadruplex-Hemin-HRRHKHRRH) surpasses the natural CAT/POD bifunctional enzyme KatG, and competes with individual CAT and POD enzymes. This remarkable performance is attributed to the strategic combination and incorporation of histidine (H) and arginine (R) residues, which effectively trap hydrogen peroxide (H_2_O_2_) near the catalytic center *via* hydrogen bond formation, thus facilitating the generation of the active intermediate compound I, as supported here by theoretical calculations. Significantly, bi-CPDzyme achieves efficient decomposition of bleaching-derived residual H_2_O_2_ in a water-/energy-saving manner, while degrading dyes from textile industry effluents even in complex real samples, in addition to being easily recyclable and implementable. These findings make bi-CPDzyme a cutting-edge and environmentally friendly catalyst, positioning it at the forefront of advancements towards creating a sustainable society.

## INTRODUCTION

The printing and dyeing industry is an economic sector with many ramifications in our daily lives. However, the related industrial processes are complex (multistep), expensive (being time-, water-, energy- and material-consuming, notably because of the boiling, bleaching, dyeing, printing and post-treatment steps) and not ecofriendly [1,2]. As an example, hydrogen peroxide (H_2_O_2_) used for the bleaching process that is performed at high temperatures and under strong alkaline conditions, must be completely decomposed before the dyeing step, which necessitates multiple harsh and energy-intensive washing procedures. Subsequently, the treatment of wastewater generated during the dyeing step also requires high water consumption to remove contaminants before being released into watercourses [3–5].

A method to eliminate H_2_O_2_ relies on the use of the catalase (CAT) enzyme [6,7], the disadvantage of which being its sensitivity to high temperature and pH [8]. In addition, the dyes used for staining, along with dye intermediates, acids, bases, by-products and several organics used all along the process, are pollutants [9,10] that necessitate efficient coping strategies like physical, chemical and biological treatments [11]: some of them are efficient but also have a cost from both financial and environmental points of view [12]. In the search for more ecofriendly catalysts, there is a growing need for greener alternatives [13]. One promising candidate is the peroxidase (POD) enzyme extracted from multiple species, which has been employed for the remediation of commercial dyes [14–16]. However, both CAT- and POD-based systems are vulnerable to extreme pH and temperatures, and unsuitable for use in organic solvents [17]. Therefore, massive efforts are being invested to develop new materials such as Fenton reactive nanomaterials [18,19] and enzyme-MOFs [20] for instance, aimed at being used as substitutes for natural enzymes; however, these synthetic systems are not devoid of issues, including low recyclability rates and possible generation of hazardous waste [21,22].

Two parameters must be fine-tuned and combined to pave the way for next-generation green catalysts: first, the enzymatic system must be usable in harsh experimental conditions found in the printing and dyeing industry (extreme pH and temperatures, practically convenient solvents); second, the CAT and POD activities must be combined to optimize the processing of high concentrations of H_2_O_2_ and wastewater treatment. Such bifunctional enzymes exist in nature, e.g. the catalase-peroxidase KatG [23,24], but they suffer from an intrinsic fragility limitation along with a rather low POD activity despite a high CAT activity [25]. More practically convenient, so-called the artificial enzymes have been reported over the past years including engineered metalloenzymes, *de novo* designed enzymes [26–28], G-quadruplex (G4)-based DNAzymes [29], along with several nanozymes (Prussian blue, Mn@Bi_2_Se_3_ among others) [30–32]. These systems usually exhibit good POD activity but relatively poor CAT activity. Recently, we proposed the concept of chimeric peptide-DNAzyme (CPDzyme): this system combines the advantages of both natural enzymes (high catalytic activity) and DNAzymes (high chemical robustness) [33]. Our first CPDzyme prototype was found to be both efficient, with a POD activity surpassing (*k*_cat_) that of the parent horseradish peroxidase (HRP), and robust, being usable in a wide range of non-physiological environments. However, the CAT activity of CPDzymes remains to be established and/or optimized.

**Figure 1.**
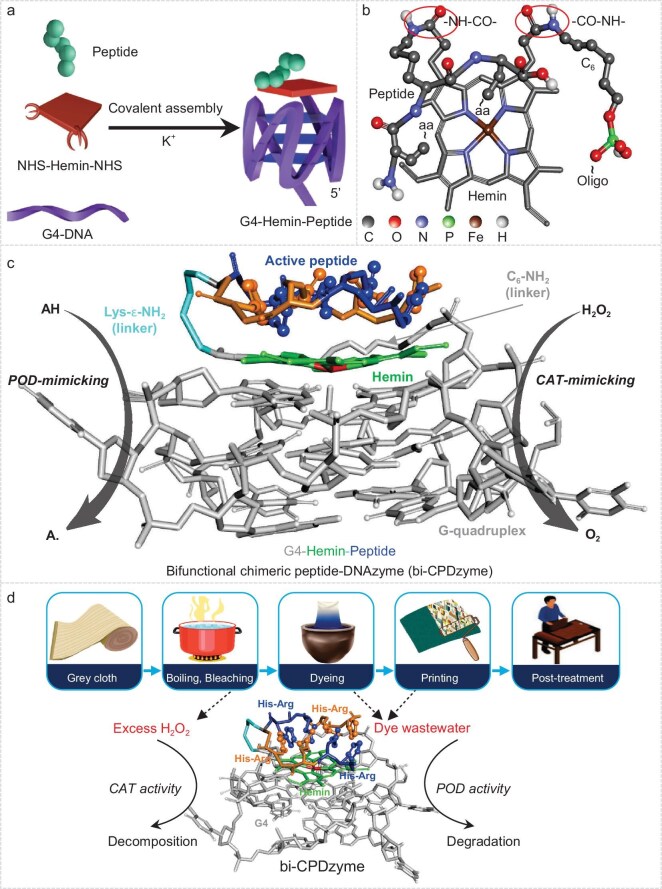
Bi-CPDzyme assembly and applications. (a) Bi-covalent assembly schematic (details in Fig. S1). (b) Hemin catalytic center with dual amide bonds (red ovals) linking DNA/peptides (aa: amino acid). (c) Schematic illustrations of the G4-Hemin-peptide, with G4 (gray), hemin (green) and active peptide (blue) highlighted, mimicking peroxidase (POD) and catalase (CAT). (d) Flowchart of the application of bi-CPDzyme in the dyeing and printing process using its CAT and POD activities.

In this context, we report here on the design and synthesis of the first CAT/POD bifunctional CPDzyme (bi-CPDzyme) system along with its use for tackling the environmental issues associated with the printing and dyeing industry. To this end, we took the CPDzyme concept a step further: we assembled a G4 DNA and a short active peptide around a hemin (chloroprotoporphyrin IX iron(III)) core (Fig. 1a), thereby benefitting from its two chemically addressable carboxylic arms which are easily converted into two amide bonds (red circles, Fig. 1b). The resulting bi-CPDzyme was found to exceed the activity of KatG, which results from the cumulative effect of multiple histidine-arginine (His-Arg) repetitions inside the active site of the artificial enzyme, as compared to a single repetition for the natural enzyme (Fig. 1c). More importantly, this system is catalytically competent under non-physiological conditions and can be recycled quite efficiently. Its potential relevance for strategic applications is demonstrated herein for both one-step bleaching (in which bi-CPDzyme decomposes H_2_O_2_ without the need for multiple washings at high temperatures or pH adjustment, thus reducing water and energy usage) and wastewater disposal (degrading dyes in complex solvent mixtures) (Fig. 1d). As further detailed hereafter, bi-CPDzyme satisfies the key performance criteria expected of a promising green catalyst, as this artificial enzymatic system is highly efficient, made of non-toxic and harmless raw materials on the basis of environment-friendly processes.

## RESULTS AND DISCUSSION

### Synthesis and characterization of bi-CPDzymes

Our artificial enzyme systems, bi-CPDzymes, were constructed (see details in the Methods section) and optimized for peptide linking (described further below). Briefly, the central hemin core (NHS-hemin-NHS) was connected to the G4 unit on one side (biotin-T_9_/iPCLink/TG_3_TG_3_TG_3_TG_3_-NH_2_, itself linked to streptavidin (STV)-coated magnetic beads (MBs), with iPCLink: internal photocleavage site), and to the peptide arms on the other side (Table S1). The iPCLink was used to free the complex from the MBs (UV irradiation) and G4 folding was ensured by the presence of potassium cations (Fig. S1). The structural integrity of bi-CPDzyme (Figs S1–S4) was demonstrated by UV and fluorescence measurement, to testify to the presence of both G4 and hemin in the bi-CPDzyme structure, along with electrospray ionization (ESI-MS) and inductively coupled plasma (ICP-MS) mass spectrometry, to characterize the whole assembly through mass/charge ratio and metallic content.

### Lessons from natural enzymes to design bi-CPDzymes

**Figure 2.**
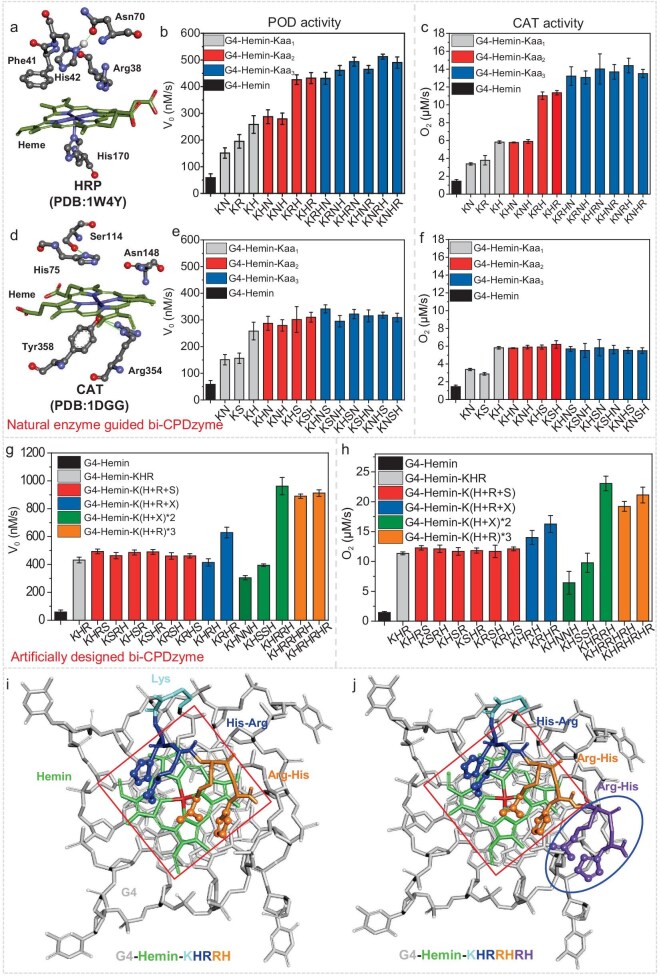
Bi-CPDzyme design and activity analysis. (a–f) Key heme-binding residues in HRP (a) and CAT (d) and their effects on POD (b, e) and CAT (c, f) activities in bi-CPDzyme. (g, h) Performance of engineered peptides (vs G4-Hemin control). (i, j) Top-view structures of G4-Hemin-KHRRH (i) and -KHRRHRH (j) systems (red box: hemin occupancy; blue oval: tertiary H + R).

Both peroxidases HRP (PDB ID: 1W4Y, Fig. 2a) and cytochrome c peroxidase (CcP, PDB ID: 4CVJ, Fig. S5a) share a remarkably conserved histidine (His42 and His52 for HRP and CcP, respectively) at the core of their active site. This histidine, along with arginine (Arg38 and Arg48 for HRP and CcP, respectively) plays a crucial role in the formation and stabilization of an intermediate compound referred to as compound I [34,35] during the catalytic reaction. In addition, an asparagine (Asn70 or Asn82 for HRP and CcP, respectively) facilitates the process by forming hydrogen bonds with this His to increase its basicity [36,37]. This amino acid network is reminiscent of both the active site of KatG and the cofactor binding region of CcP [38]. Also, each subunit of tetrameric CAT (PDB ID: 1DGG, Fig. 2d) contains its own heme and a combination of His75 + Asn148 as well as a serine (Ser114) that plays the same role as Asn in PODs (Fig. S5a) [39,40].

We exploited these amino acids' roles in the bi-CPDzyme prototypes (Fig. S5b): the effect of single/multiple amino acid(s) on the POD/CAT bifunctional activity was evaluated by the model oxidation reaction between 2,2′-azino-bis(3-ethylbenzothiazoline-6-sulfonic acid) (ABTS) and H_2_O_2_ for the POD activity, and monitoring the concentration of produced O_2_ for the CAT activity. Among the core HRP amino acids, Phe was discarded as it was shown not to affect CPDzyme activity [33]. The effects of three amino acids His, Arg and Asn (using a Lys (K) as a linker) on CPDzyme activity are summarized in Fig. 2b and c. Briefly, (*i*) for a single amino acid addition, G4-Hemin-KH showed the highest bifunctional activities, with an initial velocity of ABTS oxidation (POD-like activity) *V*_0-POD_ = 258 nM/s and an initial O_2_ production (CAT-like activity) *V*_0-CAT_ = 5.8 μM/s. This result suggests that His plays an essential catalytic role [41,42]; (*ii*) for two amino acids, the best combination was His + Arg (H + R), which was found to increase both POD- and CAT-like activities, with *V*_0-POD_ = 432 nM/s and *V*_0-CAT_ = 11.3 μM/s, respectively. This enhancement may originate in the basicity of the Arg residue (pKa = 12.5), which could activate H_2_O_2_ in coordination with His and accelerate the formation of compound I [43,44], thus making the H + R combination central to the catalysis; (*iii*) for three amino acids, no significant improvements were noticed using His, Asn and Ser combinations (Fig. 2e, f). As this first series of results demonstrated that the H + R combination provides the strongest activation, we decided to increase the number of H + R combinations (Fig. 2g, h): (*i*) both POD- and CAT-like activities benefitted from the extension of H + R tails with *V*_0-POD_ = 982 nM/s and *V*_0-CAT_ = 23.2 μM/s for G4-Hemin-KHRRH, thereby being 2.3- and 1.9-fold higher than a single H + R combination (G4-Hemin-KHR); (*ii*) the H + R tail was further extended to G4-Hemin-KHRRHRH and -KHRHRHR, but without any increase in efficiency, likely due to the steric hindrance within the active site of CPDzyme. This is illustrated in Fig. 2i and j, where the red square represents the space occupancy of hemin and the dark blue oval the third H + R repeat within peptide-KHRRHRH, which is outside of the active site.

### Design of optimized bi-CPDzymes

**Figure 3.**
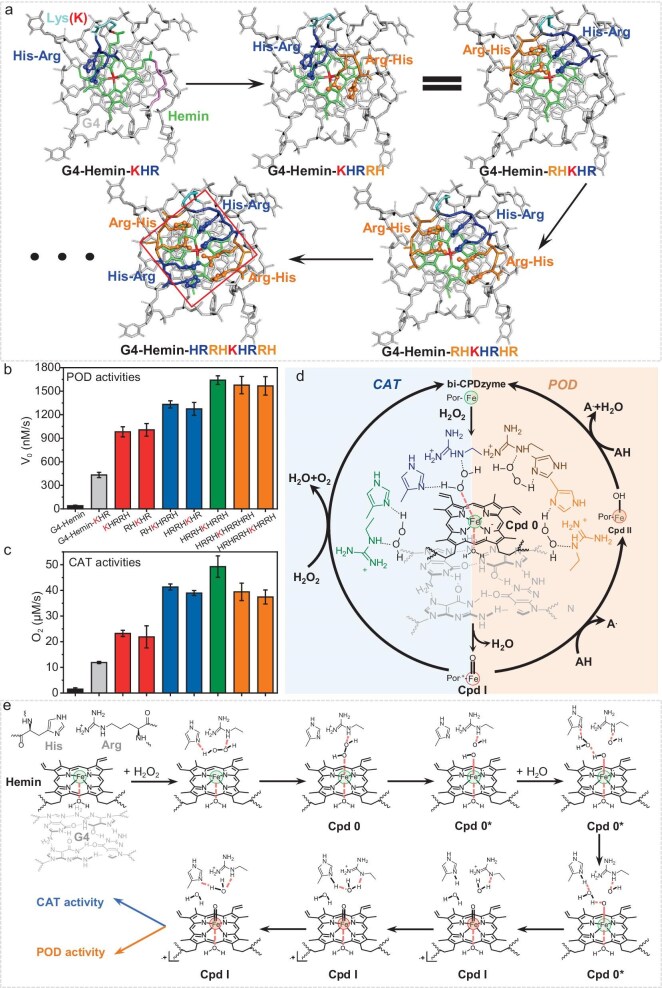
H + R pair evolution in bi-CPDzyme and catalytic mechanism. (a) Top-view structural progression from single to quadruple H + R pairs. (red box: four pairs maximize hemin’s space occupancy). (b, c) Activity assays: POD (b) and CAT (c) performance. (d) Catalytic mechanism of bi-CPDzyme (CAT: light blue; POD: orange). (e) Intermediate compound I formation (Fe(III)/Fe(IV) transition: green/red spheres).

To further benefit from the available space above the hemin binding site, the position of the Lys residue was shifted (towards the N- or C-terminal, or in the middle of the oligopeptide) and the consequence on the catalytic activity of the resulting bi-CPDzyme was monitored (Fig. 3a–c and Fig. S6): (*i*) no position-dependent effect was assessed within the HRRH tail (neither in the L- nor D-series); (*ii*) in the (H + R)_2_ tail, this effect was also weak, with *V*_0-POD_ between 982 and 1006 nM/s and *V*_0-CAT_ between 21.9 and 23.2 μM/s; (*iii*) in the (H + R)_3_ tail, a noticeable effect was obtained with HRRHKHR (or RHKHRRH) when the Lys was located at intermediate positions, with *V*_0-POD_ up to 1331 nM/s and *V*_0-CAT_ up to 38.9 μM/s, which represents a 3.1- and 3.3-fold improvement as compared to a bi-CPDzyme displaying a single H + R (*V*_0-POD_ = 432 nM/s and *V*_0-CAT_ = 11.9 μM/s); (*iv*) in the (H + R)_4_ tail, a similar improvement was obtained with HRRHKHRRH, with (*V*)_0-POD_ = 1640 nM/s and *V*_0-CAT_ = 49.3 μM/s, which represents a 3.8- and 4.1-fold improvement as compared to a bi-CPDzyme displaying a single H + R; (*v*) finally, no positive effects were seen when further extending the H + R tail to five units. This second series of results provides interesting insights into the available space within the active site and how the H + R tail length can be fine-tuned to maximize the catalytic activity of the resulting bi-CPDzymes.

### Proposed mechanism of bi-CPDzyme catalysis

A schematic representation of the bifunctional POD/CAT catalytic cycle can be seen in Fig. 3d. The generation of Por^.+^-Fe^IV^=O (compound I, Cpd I) [45], an important active intermediate in the POD/CAT catalytic cycle, is shown schematically in Fig. 3e. His-N and Arg-NH form intermolecular hydrogen bonds with H_2_O_2_ and then the H + R coordinates H_2_O_2_ delivery to the Por-Fe^III^ center to form compound 0 (Cpd 0). Subsequent O–O bond cleavage generates Cpd 0* and the free OH^−^ forms a hydrogen bond with Arg-NH. Through the mediation of a single H_2_O molecule, the proton is transferred from Fe–O–H to His-N to regenerate Cpd I. The EPR spectrum of Cpd I harbors an asymmetrical signal with a *g*-value of 1.997 at 77 K (Fig. S7), which is similar to that of HRP (*g* = 1.995, 4.2 K) [46]. Meanwhile, OH^−^ attracts a proton from Arg to form stable H_2_O, which is then exchanged with the protonated His to regenerate the initial Arg-NH/His system. In the absence of a substrate, Cpd I reacts with a second molecule of H_2_O_2_ to produce O_2_ and H_2_O, the bi-CPDzyme thus exhibiting a CAT activity [41,47]. In the presence of a substrate (AH), Cpd I is reduced to Por-Fe^IV^=OH (Cpd II) and produces the radical A^.^; a second AH molecule reduces Cpd II to the native bi-CPDzyme Fe^III^ and produces a second radical A^.^, the bi-CPDzyme thus exhibiting a POD activity. This mechanism highlights the positive and coordinated contributions of the H + R pairs, found to improve both the speed and efficiency of the catalysis. The proposed mechanism here was supported by the cumulative effect of H + R pairs: the catalytic activity of the bi-CPDzyme system does indeed positively correlate with the number of H + R pairs (for (H + R)_≤4_, Fig. 3b, c). This observation indicates that the H + R pairs help capture H_2_O_2_ and accelerate the formation of Cpd I, which are the two important steps in the POD/CAT-catalyzed reaction. With (H + R)_≥4_, such a cumulative effect is lost, presumably due to the introduction of steric hindrance within the active site (see Fig. 2j).

### The roles of H + R pair in catalysis

**Figure 4.**
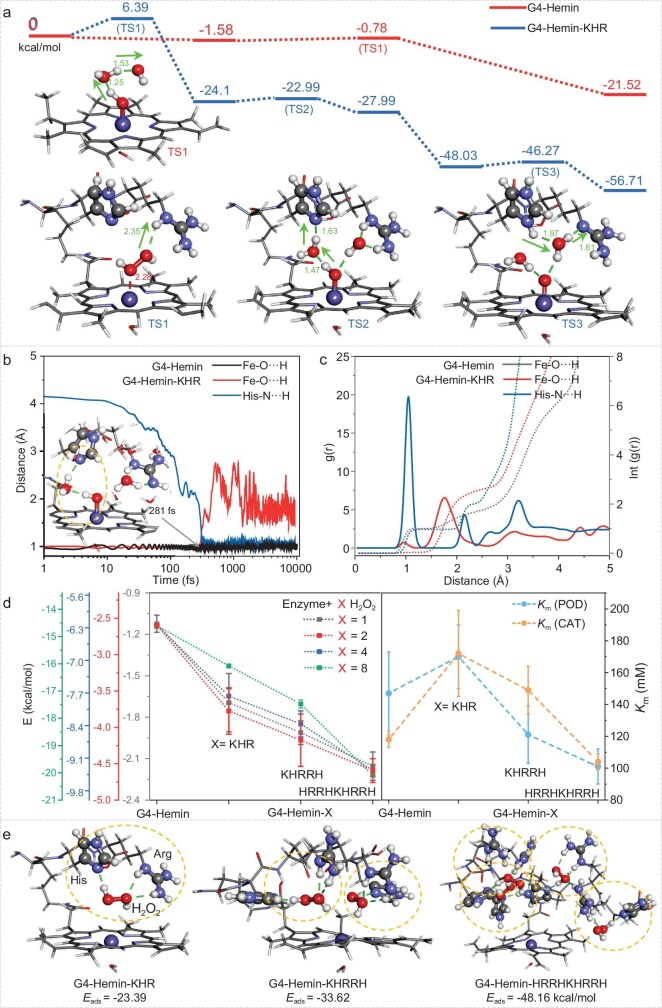
The proposed catalytic processes mediated by amino acids. (a) Energetic diagram of possible reaction intermediates and transition states (TS) during the generation of compound I by G4-Hemin and G4-Hemin-KHR. (b) Evolutions of the distance between the Fe–O/His-N and the H and (c) the pair distribution functions of O/N and H atoms. (d) Binding energies of G4-Hemin and bi-CPDzymes with one, two, four and eight H_2_O_2_, and *K*_m_ values (POD/CAT) from Table 1. (e) Adsorption energy (*E*_ads_) of H_2_O_2_ to binding sites in the cavity of bi-CPDzyme.

Density functional theory (DFT) calculations were performed to map the formation of Cpd 0 and Cpd I. In the ‘simple’ G4-Hemin system, H₂O₂ first binds to Por-Fe^III^ to give Cpd 0; subsequent O–O cleavage furnishes Cpd 0*, whose proton transfer to Cpd I plus two H_2_O molecules proceeds via TS1 (Δ*G*‡ = 0.80 kcal/mol, red, Fig. 4a, Fig. S8a and Movie S1). Despite this low barrier, kinetic trapping of H_2_O_2_ is hindered because surrounding bulk water can hydrogen-bond with the peroxide and delocalize it away from the iron center. In the G4-Hemin-KHR system, this issue is alleviated as His-N and Arg-NH together with the iron center pre-organize H_2_O_2_ and its fragments. Their concerted action lowers kinetic dispersion: cleavage proceeds through TS1 (Δ*G*‡ = 6.39 kcal/mol, blue, Fig. 4a and Fig. S8b) that simultaneously involves Fe and Arg-NH, after which the nascent OH⁻ is captured by Arg-NH and protonated to yield H_2_O. Accordingly, G4-Hemin relies on a single TS1 in which a water molecule shuttles one proton. In G4-Hemin-KHR the process is split: TS2 (Δ*G*‡ = 1.11 kcal/mol) moves a proton from Fe–OH to His-N via water, and TS3 (Δ*G*‡ = 1.76 kcal/mol) relays the proton from His-NH⁺ to Arg-N, again through a bridging water, to complete the cycle (Movie S2). Although the overall barrier for Cpd I formation is higher in G4-Hemin-KHR than in G4-Hemin (6.39 vs 0.8 kcal/mol), the amino acids restrict H_2_O_2_ and its fragments to the iron center, kinetically promoting the Por-Fe^III^/H_2_O_2_ encounter.

Next, we calculated the fate of Cpd 0* in both G4-Hemin and G4-Hemin-KHR systems with *ab initio* molecular dynamics (AIMD). The monitored O–H distance of Fe–OH in the G4-Hemin system fluctuates around 1 Å (black line, Fig. 4b), indicating that the proton is hardly removed to generate Cpd I. The cleaved OH⁻ drifts among bulk water molecules and away from Fe (Fig. S9, Movie S3), underscoring the sluggish Cpd 0* → Cpd I step. In G4-Hemin-KHR, this O–H distance rises and fluctuates around 2 Å (after 281 fs) while the N–H distance of His-N decreases to 1 Å (red and blue lines, Fig. 4b). The derived pair distribution functions (PDFs) for His-N show a distinct peak centered at 1 Å (solid lines, Fig. 4c) with an integral of 1 each (dashed lines, Fig. 4c), confirming N–H formation. The Fe–O PDF, however, has only a weak 1 Å feature integrating to ∼0, evidencing successful Cpd I generation. In G4-Hemin, the Fe–O PDF shows a single 1 Å peak integrating to 1, confirming that no OH⁻ remains near the iron and the proton is never abstracted. Taken together, AIMD snapshots (Fig. S10) reveal that only in G4-Hemin-KHR can the proton shuttle smoothly from Fe–O to His-N. Moreover, the free OH⁻ exhibits an oxygen coordination number of 2, while Arg-N1 shows 1, indicating a hydrogen bond between OH⁻ and Arg-N1···H (Fig. S11). These results corroborate the DFT pathway for Cpd 0* → Cpd I in G4-Hemin-KHR (Fig. S8b, Movie S4). The results of these calculations reveal the catalytic importance of the H + R pair: the hydrogen-bonds form between guanidinium group of Arg and H_2_O_2_, which can stabilize Cpd 0 (Por-Fe^III^–H_2_O_2_, TS1) to facilitate O–O bond cleavage. The guanidinium group subsequently captures the nascent OH⁻ *via* hydrogen bonding, spatially constraining this proton-accepting species near the hemin center. Through a bridging water molecule, His mediates proton abstraction from Fe-OH, driving Cpd I (Por^.+^-Fe^IV^=O) formation. Then, the confined OH⁻ extracts a proton from the guanidinium group to form H_2_O, which ultimately participates in proton transfer with the protonated His, regenerating the catalytically competent H + R pair.

We then further characterized the advantages of multiple H + R pairs in bi-CPDzyme by calculating the electrostatic potential (ESP): this revealed that the positive charge increased with the number of H + R pairs, which is favorable to electrostatically attract the O atom of H_2_O_2_ (Fig. S12). Furthermore, the binding energies (docking) of bi-CPDzymes to one, two, four and eight H_2_O_2_ molecules progressively decrease as the number of H + R pairs increase from 0 to 4 (Figs S13 and S14), implying an increase in affinity of bi-CPDzyme for H_2_O_2_, as supported by the *K*_m_ value measured for the POD/CAT activity (Fig. 4d). Finally, the adsorption energies (*E*_ads_) of H_2_O_2_ to the binding sites of bi-CPDzyme were evaluated by DFT and we found that one, two and four H + R pairs could adsorb one, two and four H_2_O_2_ molecules with *E*_ads_ of −23.39, −33.62 and −48.16 kcal/mol, respectively (Fig. 4e). Therefore, the G4-Hemin-HRRHKHRRH system seems to be optimized for binding to multiple H_2_O_2_ molecules, thus increasing the probability of the enzyme binding to H_2_O_2_ and the reaction between hemin and H_2_O_2_.

### Kinetic analysis of bi-CPDzymes catalysis

To gain more accurate insights into the catalytic performance of bi-CPDzymes, their steady-state kinetics were analyzed (Table 1 and Figs S15–S17). The POD and CAT turnover numbers (*k*_cat_) of G4-Hemin were used as references, with *k*_cat_ = 33.4 s^−1^ and 482 s^−1^, respectively. The *k*_cat_ of G4-Hemin-KHR reached 430 s^−1^ (POD) and 1820 s^−1^ (CAT). Significant improvements were observed with two H + R pairs (G4-Hemin-KHRRH and G4-Hemin-RHKHR showed roughly the same kinetic parameters) while G4-Hemin-HRRHKHRRH provided the best results, with *k*_cat_ = 1245 s^−1^ (POD), which is ∼2.4-fold of the natural enzyme HRP (*k*_cat_ = 521 s^−1^) [33], and *k*_cat_ = 6341 s^−1^ (CAT), which is ∼0.77-fold of natural CAT (the *k*_cat_ of the natural CAT is 32 891 s^−1^, and reported *k*_cat_ in the literature ranges from 9628 to 74 800 s^−1^ [48,49], but it should be noted that natural CAT is a tetramer, the *k*_cat_ value for a single active center is thus 8223 s^−1^) (Fig. S18). Interestingly, G4-Hemin-HRRHKHRRH outcompetes the bifunctional KatG enzyme (with *k*_cat_ = 7.7–25 s^−1^ (POD) and 2950–7770 s^−1^ (CAT)) [25], thus being 50- to 162-fold (POD) and 0.8- to 2.1-fold more active (CAT) than KatG, and also offers significant advantages over other reported bifunctional artificial enzymes (Table S2). The Michaelis constant (*K*_m_) of bi-CPDzymes decreases with the increase in the number of H + R pairs (from 170 to 101 mM (POD) and 172 to 104 mM (CAT)), which corresponds to an increase *k*_cat_/*K*_m_ value, from 2.53 to 12.3 s^−1^ mM^−1^ (POD) and 10.6 to 61.0 s^−1^ mM^−1^ (CAT). The *K*_m_ value being higher than that of natural enzymes, in which the unique substrate channel actively participates in catalysis. Of note, the high H_2_O_2_ concentrations used in routine experimental setups make the *k*_cat_ value more important than *K*_m_; therefore, the excellent *k*_cat_ of bi-CPDzymes make them very promising for practical applications.

**Table 1.** Kinetic parameters of POD and CAT reactions catalyzed by bi-CPDzyme.

		*K*_m_^a^ (mM)	*V*_max_^b^ (μM·s^−1^)	*k*_cat_^c^ (s^−1^)	*k*_cat_/*K*_m_ (s^−1^·mM^−1^)
G4-Hemin	POD	147 ± 26	0.167 ± 0.018	33.4 ± 3.6	0.20 ± 0.05
	CAT	118 ± 5	4.81 ± 0.09	482 ± 9	4.1 ± 0.26
G4-Hemin-KHR	POD	170 ± 20	2.149 ± 0.153	430 ± 31	2.53 ± 0.54
	CAT	172 ± 27	18.2 ± 1.27	1820 ± 127	10.6 ± 2.85
G4-Hemin-KHRRH	POD	121 ± 18	4.191 ± 0.355	838 ± 71	6.92 ± 1.90
	CAT	149 ± 15	41.03 ± 1.71	4103 ± 171	27.5 ± 4.36
G4-Hemin-RHKHR	POD	120 ± 17	4.003 ± 0.318	801 ± 64	6.68 ± 1.72
	CAT	153 ± 17	36.67 ± 1.81	3667 ± 181	24.0 ± 4.33
G4-Hemin-HRRHKHRRH	POD	101 ± 11	6.226 ± 0.376	1245 ± 75	12.3 ± 2.37
	CAT	104 ± 5	63.41 ± 1.15	6341 ± 115	61.0 ± 4.24

a [H_2_O_2_] mM; ^b^POD kinetic parameters measured by 5 mM ABTS oxidation; ^c^POD and CAT reactions catalyzed by 5 and 10 nM bi-CPDzyme.

### Chemical stability of bi-CPDzymes

The printing industry makes most frequent use of redox reactions under conditions not suited to the use of natural enzymes (high temperatures, strongly acidic and basic conditions, organic solvents, etc.). As CPDzymes were previously shown to be highly robust in harsh conditions [33], we decided to assess the activity of bi-CPDzymes under these conditions: the results seen in Figs S19 and S20 demonstrate the operability of G4-Hemin-HRRHKHRRH: (i) it works within a wide pH range of 2 to 12; for instance, neutral and weakly acidic conditions favor POD-like activity, and CAT-like activity exhibits higher tolerance between pH 4 and 10 (Fig. S19a). (ii) It shows the robustness in solutions containing 50% (v/v) of various organic solvents, retaining 25%–57% of its POD-like activity and 58%–92% of its CAT-like activity relative to buffer solutions (Fig. S19b). (iii) It displays excellent activity under thermal stress: after a 1-h treatment at 95°C, the POD-like and CAT-like activities are 86% and 57% maintained, respectively (Fig. S19c). (iv) It preserves >95% of its initial activity after 21 days of storage at room temperature (Fig. S20a). (v) It exhibits high reusability, evidenced by maintaining >90% activity after 10 catalytic cycles when immobilized on MBs (Fig. S20b). Collectively, these outstanding performances highlight the usability potential of bi-CPDzymes under actual complex conditions.

### Applications of bi-CPDzymes

The ultimate application of bi-CPDzymes lies in their ability to decompose H_2_O_2_ and decontaminate dye wastewater originating from the printing industry. As schematically represented in Fig. 1d, the industrial boiling and bleaching steps require the use of both an excess of H_2_O_2_ at high temperatures and strong alkaline conditions. The CAT-like activity of bi-CPDzyme is intended to decompose H_2_O_2_ into H_2_O and O_2_ while its POD-like activity is intended to degrade the contaminants found in dye wastewater.

**Figure 5.**
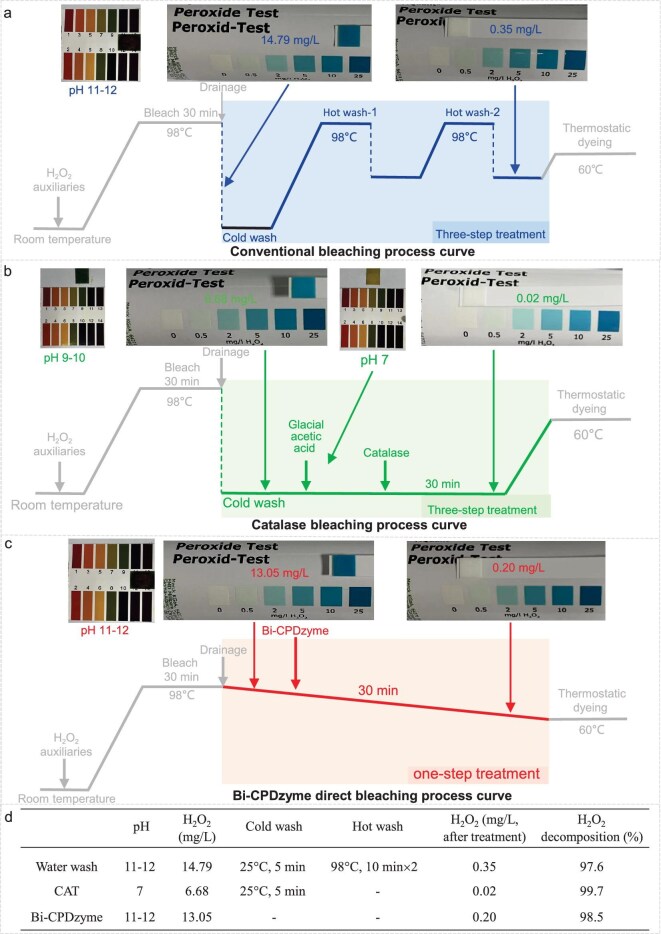
Water-saving application of bi-CPDzyme. (a) The conventional washing and (b) natural catalase bleaching process. (c) Direct bleaching process curve of bi-CPDzyme. (d) Essential steps and H_2_O_2_ decomposition rates (%) for three bleaching processes.

We first implemented the bleaching post-treatment process seen in Fig. 5, Figs S21 and S22: to this end, a piece of textile was bleached by treatment with H_2_O_2_ auxiliaries, the excess H_2_O_2_ was removed by either washing steps or enzyme/bi-CPDzyme treatment, and the residual H_2_O_2_ was quantified by H_2_O_2_ test strips. The conventional high-temperature washing process involves the addition of H_2_O_2_ at 25°C followed by a heating step (30 min at 98°C), a drainage step (the pH measured after the drainage is between 11 and 12; the concentration of H_2_O_2_ is 14.79 mg/L) and three washings, one with cold water (25°C for 5 min), and two with hot water (98°C for 10 min) (Fig. 5a). At the end of this tedious process, the final concentration of H_2_O_2_ was 0.35 mg/L, and the decomposition rate was 97.6%. The enzymatic washing process using CAT was adapted from the previous approach; however, as CAT is inactivated under alkaline conditions, glacial acetic acid was added to adjust the pH of the solution to pH 7 during the process (Fig. 5b), which led to an H_2_O_2_ decomposition rate of 99.7%. When using bi-CPDzyme G4-Hemin-KHRRHKHRRH at the same concentration as CAT (for 30 min at pH 11–12) directly without washings, the H_2_O_2_ decomposition rate was 98.5% (Fig. 5c). The results, summarized in Fig. 5d, indicate that using bi-CPDzyme allows for removing the steps of hot/cold water washings and pH adjustment, while being equally efficient. Therefore, the one-step treatment with bi-CPDzyme improves the bleaching process in a time-, water- and energy-saving manner.

**Figure 6.**
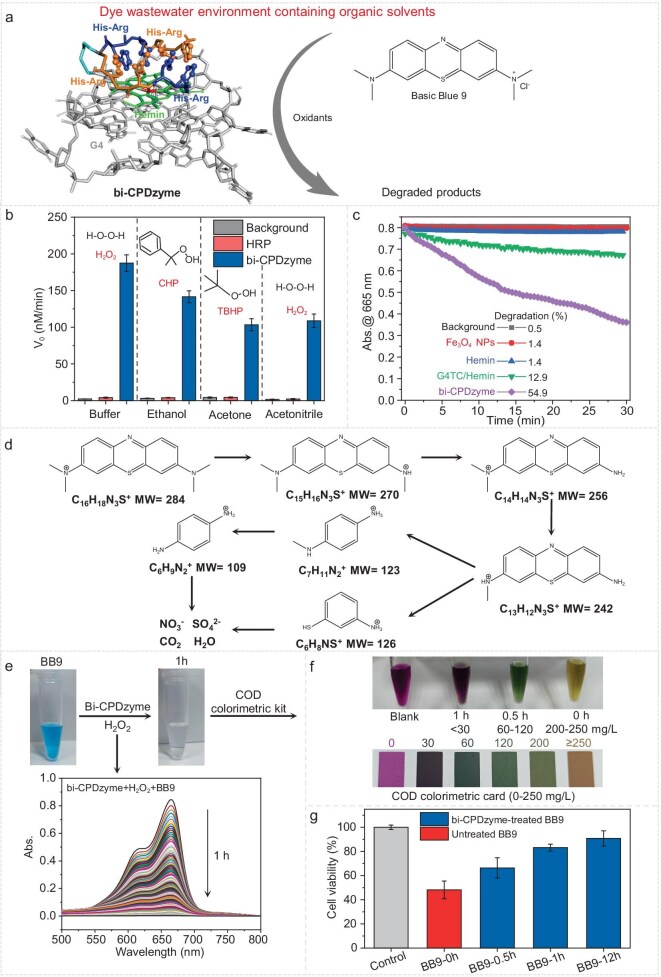
Comprehensive dye wastewater treatment by bi-CPDzyme. (a) BB9 degradation schematic with oxidants/organic solvents. (b) Comparative degradation (1 mM oxidants) in solvents (HRP vs bi-CPDzyme). (c) UV absorption kinetics (1 mM H_2_O_2_) with artificial catalysts. (d) Possible degradation pathway of BB9 catalyzed by bi-CPDzyme. (e) Visual/spectral changes after 1 h treatment. (f) COD removal efficiency over time. (g) HEK293T cell viability assessment.

Next, we focused on wastewater decontamination: the ability of G4-Hemin-KHRRHKHRRH to degrade Basic blue 9 (BB9), a popular dye used in industry (textiles, rubber, plastics, cosmetics, etc.), was chosen as a model reaction. Of note, as we aimed at working in different water/organic mixtures, we also selected different oxidants, soluble in the experimental conditions (Fig. 6a). The performance of bi-CPDzyme was directly compared to that of HRP: as seen in Fig. 6b and Fig. S23, when using H_2_O_2_ at pH 7, the catalytic rate of HRP was 4.1 nM/min and that of bi-CPDzyme 187.4 nM/min, thus being ∼46-fold higher than HRP. The catalytic rate of bi-CPDzyme reached 141.5 nM/min in 50% ethanol (v/v) using cumyl hydroperoxide (CHP) as an oxidant, 103.4 nM/min in 50% acetone (v/v) using tert-butyl hydroperoxide (TBHP) as an oxidant, and 108.8 nM/min in 50% acetonitrile (v/v) using H_2_O_2_ as an oxidant, all these conditions being unsuited to the use of HRP.

We then compared the degradation rate of BB9 by bi-CPDzyme with two artificial catalysts, i.e. Fe_3_O_4_ nanoparticles (NPs) and G4TC/Hemin DNAzyme: as seen in Fig. 6c, Figs S23 and S24, under the same molar concentration of catalysts, the degradation rate of Fe_3_O_4_ NPs was 1.4%, that of G4TC/Hemin 12.9%, far behind that of bi-CPDzyme (54.9%) in 30 min, demonstrating the superiority of bi-CPDzyme among these artificial catalysts. We also investigated the byproducts generated during BB9 degradation in order to assess whether some of them could be harmful. As shown in Fig. S25a, the BB9 solutions changed from blue to colorless when they were treated by bi-CPDzyme with 0.3 to 300 mM H_2_O_2_ concentration for 12 h, indicating the total disappearance of the chromogenic groups, which was confirmed by both UV measurements (Fig. S25b) and high-performance liquid chromatography (HPLC) (Fig. S26). Then, the degradation products were identified by liquid chromatography-mass spectrometry (LC-MS): several peaks were seen corresponding to several degradation intermediates (details in Fig. S27), with the terminal degradation products found to be NO_3_^−^ and SO_4_^2−^, as detected by ion chromatography (IC) (Fig. S28). We thus showed that the degradation products of BB9 by bi-CPDzyme (Fig. 6d) include nontoxic inorganic ions, carbon dioxide and water. To go a step further, we assessed the toxicity of BB9 before and after degradation: after 1 h of treatment by bi-CPDzyme, the BB9 sample changed from blue to colorless, the absorption peak at 665 nm decreased from 0.85 to 0.002 (Fig. 6e), and the corresponding chemical oxygen demand (COD) value decreased from 200–250 mg/L to <30 mg/L, indicating the reduction of water pollution (Fig. 6f). In addition, the HEK293T cell viability was measured to evaluate the cytotoxicity of the degradation products. The cell viability increased from 47% (control) to 66%, 83% and 91% after 0.5, 1 and 12 h of degradation, respectively (Fig. 6g). Moreover, the cell viabilities range from 62% to 73% in six degradation intermediates (Fig. S29), suggesting that toxicity was reduced after treatment with bi-CPDzyme.

Finally, we investigated the functions of bi-CPDzyme in real-life examples, that is, in textile dyeing wastewater samples: two samples were taken in a textile dyeing wastewater treatment plant—one from the primary clarifier and one from a sequencing batch reactor (SBR) (Fig. S30a, b). The maximum absorption peak at 228 nm decreased for both samples after treatment with bi-CPDzymes, indicating that pollutants with conjugate absorption were degraded (Figs S30c and S31). In addition, the COD values of the two samples decreased to 60 and 30 mg/L (76% and 75% reduction, respectively) after treatment with bi-CPDzyme, and the amount of residual H_2_O_2_ was ∼2 mg/L, indicating that the decomposition rate of H_2_O_2_ was >92% (Fig. S32a), and increased cell viability by 28.5% and 25% (Fig. S32b). Moreover, bi-CPDzyme retained 87% CAT activity and 88% POD activity after 7 consecutive catalytic cycles (Fig. S32c). These results thus demonstrate the high efficiency, stability, and reusability of bi-CPDzyme in real-life conditions, being able to catalytically degrade dyes and H_2_O_2_ in a wide range of experimental conditions. Specifically, bi-CPDzyme exhibits outstanding cost-effectiveness (21.8% of commercial enzymes, Tables S3–S5), maintains >87% activity after 7 operational cycles, and reduces wastewater toxicity by 25%–28.5%. Its exceptional environmental tolerance and reusability further support its potential for industrial-scale applications, pending future techno-economic analyses (TEAs) and pilot-scale validation.

## CONCLUSION

We report here on a bifunctional chimeric peptide DNAzyme (bi-CPDzyme) system synthesized in a simple one-step process from non-toxic and non-hazardous peptides, nucleic acids and hemin, which displays both catalase (CAT) and peroxidase (POD) activities. The catalytic performance of the optimized bi-CPDzyme (G4-Hemin-KHRRHKHRRH) prototype is superior to the corresponding natural bifunctional enzyme KatG and competes with individual CAT and HRP enzymes. Theoretical calculations reveal that this exceptional performance is due to the strategic incorporation of histidine (H) and arginine (R) residues in the binding sites of the catalytic system, which trap and maintain hydrogen peroxide (H_2_O_2_) near the catalytic center, thereby favoring the catalytic process. Importantly, the chemical robustness of bi-CPDzymes make them operatable in harsh experimental conditions (organic solvent mixtures, high temperatures, high/low pH, etc.), as demonstrated here with real-life samples, in which native enzymes cannot be used. We thus demonstrate the strategic interest of bi-CPDzymes, found to be efficient catalysts for treating printing wastewaters, decomposing both residual H_2_O_2_ and dyes still present in wastewaters, even after several hard washing steps. Compared with the traditional process, bi-CPDzyme not only shortens but also improves the workflow, which results in a decrease in the amount of water used. We are thus convinced that bi-CPDzymes represent a new class of artificial catalysts suited to a wide range of wastewater treatment processes, which can be furthermore easily recycled, thus decreasing both the cost *per se* and the environmental cost of the printing and dyeing industry. These artificial enzymes can undoubtedly be considered as next-generation green catalysts, fully in line with the objectives defined by the United Nations, as sustainable industrial development has been incorporated as Goal 9 in the 2030 Agenda for Sustainable Development (https://sdgs.un.org/goals/goal9).

## Supplementary Material

nwaf447_Supplemental_Files
