# Supplementary material for: Designing an ecofriendly catalyst for a sustainable use of water resources
Source: Natl Sci Rev. 2025 Oct 20;13(2):nwaf447. doi: 10.1093/nsr/nwaf447 (PMC12860202; doi:10.1093/nsr/nwaf447)
Supplement: nwaf447_Supplemental_Files [file nwaf447_supplemental_files.zip › 1262-Supporting Information-updated.pdf]

Supporting Information

Designing an Ecofriendly Catalyst for a Sustainable Use of Water Resources

Dehui Qiu[†], Xiaobo Zhang[†], Fan Tian[‡], Yuan Liu[†], Fangni He[†], Xinrong Yan[†], Shijiong Wei[†], Jean-Louis Mergny[†,§], David Monchaud[⊥], Shujuan Zhang[#], Huangxian Ju[†], and Jun Zhou[†,*]

[†]State Key Laboratory of Analytical Chemistry for Life Science, School of Chemistry & Chemical Engineering, Nanjing University, Nanjing 210023, China.

[‡]Key Laboratory of Green Chemical Process of Ministry of Education, School of Chemistry and Environmental Engineering, Wuhan Institute of Technology, Wuhan 430205, China.

[§]Laboratoire d'Optique et Biosciences (LOB), Ecole Polytechnique, CNRS, INSERM, Institut Polytechnique de Paris, 91120 Palaiseau, France.

[⊥]Institut de Chimie Moléculaire de l'Université de Bourgogne (ICMUB), CNRS UMR6302, Université Bourgogne Europe (UBE), 21078 Dijon, France.

[#]State Key Laboratory of Pollution Control and Resource Reuse, School of the Environment, Nanjing University, Nanjing, China.

*Corresponding Authors. E-mail: jun.zhou@nju.edu.cn.

Supporting Information Text

Materials and reagents

HPLC purified DNA and peptides, listed in **Table S1**, were purchased from Sangon Biotech (Shanghai, China) without further purification. Dimethyl sulfoxide (DMSO), hemin, horseradish peroxidase (HRP), catalase (CAT), cumyl hydroperoxide (CHP), tert-butyl hydroperoxide (TBHP), 2,2′-azino-bis (3-ethylbenzothiazoline-6-sulfonic acid) (ABTS), KCl, NaCl, NaOH, KOH, HCl, and Tris(hydroxymethyl)aminomethane (Tris), Thioflavin T (ThT), N-methyl mesoporphyrin IX (NMM), basic blue 9 (BB9), were obtained from Sigma-Aldrich (USA). Hydrogen peroxide (30% H_2O_2), glacial acetic acid, phosphoric acid, boric acid, acetic acid, methanol, ethanol, acetone, formamide, acetonitrile, N,N-Dimethylformamide (DMF) and 4-Dimethylaminopyridine (DMAP) were bought from Aladdin Ltd (Shanghai, China). NHS-Hemin-NHS, NH_2-DOTA-Gd and 20nm F_3O_4 nanoparticles (NPs) were ordered from Xi'an Ruixi Biological Technology Co. Ltd. (Xi'an, China). Magnetic bead (MB) modified with streptavidin were purchased from Thermo Fisher.

Hydrogen peroxide test strips were purchased from merck (Germany). All other chemical reagents with analytical grade were used directly without further purification.

Supplementary methods

Preparation of bi-CPDzyme. The preparation method is same than the previously reported synthesis [1]; briefly, as schematically represented in **Figure S1**, streptavidin-coated magnetic beads (MBs) and 10-fold excess of biotinylated DNA strands were mixed in 10 mM B&W buffer (pH 7.0, 500 μM EDTA, 1 mM K^+) and stirred at 25 ℃ for 3 hours. MBs were then isolated by magnetic separation, rinsed (thrice), resuspended in 10 mM HEPES (pH 7.0) buffer and added to a 100-fold excess of premixed NHS-Hemin-NHS, peptide, and 0.2 mg/ml DMAP (the volumes of both the aqueous and organic phases were adjusted to reach a 1:1 ratio) and the mixture was stirred at 0 ℃ overnight. Magnetic separation (as above) allowed for the isolation of the MB-G4-Hemin-peptide conjugates, which were irradiated with ultraviolet light for 30 min to free the bi-CPDzyme (photocut molecular breakage).

Measurement peroxidase (POD) and catalase (CAT) catalytic activity of bi-CPDzyme. For the measurement of POD activity, bi-CPDzymes, ABTS (1 mM) and H_2O_2 (1 mM) were mixed in 10 mM Tris-HCl buffer (pH 7.0, 100 mM K^+). The catalytic activity measurement was followed by monitoring the absorbance at 420 nm of the oxidized ABTS ($ABTS^{\cdot +}$, extinction coefficient is 36,000 $M^{-1}cm^{-1}$) using a Cary3500 (Agilent) spectrophotometer for 60 s at 25 ℃. The initial rate ($V_{0\text{-POD}}$, nM/s) of the oxidation reaction was obtained from the slope of the initial linear portion (the first 5 s) of the plot of absorbance *versus* reaction time. For CAT activity, bi-CPDzymes and H_2O_2 (100 mM) were mixed in 10 mM Tris-HCl buffer (pH 7.0, 100 mM K^+). The CAT activity was monitored by a NeoFox oxygen analyser (Ocean optics) through the concentration of oxygen produced by the bi-CPDzyme in the presence of H_2O_2. The initial rate of oxygen generation ($V_{0\text{-CAT}}$, μM/s) was

derived from the slope of the signal value of oxygen concentration *versus* the initial linear portion (first 5 seconds) of the reaction time plot. All kinetic results were obtained from triplicate experiments.

Kinetic Analysis. The oxidation reaction kinetics were established using a steady-state assay, with initial reaction rates varying the concentrations of substrates. The kinetic parameters were calculated according to the Michaelis−Menten equation: $V_0 = (V_{max} * [S]) / (K_m + [S])$, where V_0 is the initial reaction rate, V_{max} is the maximum reaction rate, [S] is the concentration of substrate, and K_m is the Michaelis constant. $k_{cat} = V_{max} / [E0]$ is the turnover number, in which [E0] is the concentration of the catalyst.

The bleaching treatment process. The piece of fabric (5*5cm) was added to a mixture of H_2O_2-auxiliaries (bath ratio = 1:20) at room temperature before being heated to 98 ℃ for 30 min. After draining the waste solution, pH and hydrogen peroxide residues on the surface of the textile were measured using pH and hydrogen peroxide test papers. The residual H_2O_2 on the fabric was removed by washing or enzyme treatment, and finally H_2O_2 residues were monitored by reading RGB values from H_2O_2 test strips in combination with mobile phone photos.

Dye wastewater degradation. The degradation rate of the dye under different conditions was determined in UV kinetic scanning mode (time interval 30 s), with a wavelength range from 500 to 800 nm. 12 μM of BB9, 1 mM oxidizing agent and 100 nM enzymes were added to 10 mM Tris-HCl buffer (pH 7.0, 100 mM K^+) containing 50% organic solvents (v/v), and UV spectra were collected at 25 ℃ for 30 min.

G-quadruplex ligands fluorescence spectra. ThT and NMM fluorescence were measured by a FS980 fluorescence spectrometer (Edinburgh Technology Limited, UK) to confirm the formation of G-quadruplex structure in bi-CPDzyme, the fluorescence enhancement of ThT and NMM ligands after binding to bi-CPDzyme. The experiments were performed using 1 μM ligands and 100 nM bi-CPDzyme in 10 mM Tris-HCl (pH 7) with 100 mM K^+ at 25 ℃.

UV-visible spectrophotometer and inductively coupled plasma-mass spectrometry (ICP-MS) quantification. The molar concentration of bi-CPDzyme was quantified by both UV (**Figure S1c**) and ICP-MS (**Figure S4**). (1) Quantification was performed using the UV absorption of hemin in bi-CPDzyme (maximum absorption wavelength of 404 nm, extinction coefficient of 146,000 $M^{-1}cm^{-1}$); (2) The G4-Hemin-DOTA-Gd samples used for the ICP-MS experiments were prepared according to the bi-CPDzyme preparation method described in the text, with the exception that the peptide in the bi-CPDzyme was replaced by NH_2-DOTA-Gd. The resulting G4-Hemin-DOTA-Gd sample was digested with nitric acid and then quantified by ICP-MS for Gd content.

Computational Details. Density functional theory (DFT) calculations were performed using the CP2K package (version 2022.1) [2]. The wave functions were expanded in molecular optimized double-ζ Gaussian basis sets (DZVP- MOLOPT-GTH) with an auxiliary plane-wave basis set with a cutoff energy of 350 Ry and rel_cutoff energy of 45 Ry. Core electrons were

modelled by scalar relativistic norm-conserving pseudo potentials with valence electrons of 16, 6, 4, 5 and 1 for Fe, O, C, N and H, respectively. The DFT-D3 van der Waals correction by Grimme was applied to describe the weak interaction in the system. Brillouin zone integration was performed with a reciprocal space mesh consisting of only the gamma point. Transition states are obtained by nudged elastic band (NEB) calculations. G4 structures have been simplified.

Ab initio molecular dynamics (AIMD) were implemented at 1 fs per step under 300K with NVT ensemble. A heating bath with the Nosé−Hoover method was employed to control the temperature of the system. The convergence criterion for the maximum force of the configuration was 4.5 x 10^{-6} hartree/Å. The energy convergence criterion of the DFT calculation in the AIMD was 3 x 10^{-5} hartree. Geometry optimizations were performed with DFT energy convergence criterion of 3 x 10^{-7} hartree until the forces acting on each atom bellowing 0.00025 eV/Å by using the wavelet Poisson solver with Broyden−Fletcher−Goldfarb−Shanno (BFGS) algorithm. All the calculations were performed under the periodic boundary conditions switched to off. The calculated results were visualized with Mercury 4.2.0 or VMD 1.9.4 packages [3].

Electrostatic potential (ESP) analysis. Geometry optimization of minima and transition state, frequency analysis, and generation of intrinsic reaction coordinate (IRC), were all performed using B3LYP functional with the 6-31G* and SDD mix basis set using Gaussian 16 program [4]. Plotting molecular structures and ESP diagram were realized by VMD 1.9.4. ESP diagrams are drawn using the default drawing method of Multiwfn 3.8(dev) software [5-7], where the wave function information is obtained from the output file of Gaussian 16 program. The results of ESP calculations are visualized by VMD software to a molecular van der Waals (vdW) surface, which is usually defined as isosurface of electron density of 0.001 a.u.

Molecular docking and analysis. AutoDock Vina software [8,9] was utilized in all docking experiments, with the optimised model serving as the docking target. In order to adapt to the universality of the docking range, we set up a docking box with a larger space including the range of the acceptor cavity for the docking. The coordinates for the docking were X: 20.673, Y: 17.852, Z: 17.526, with dimensions X: 40.0 Å, Y: 40.0 Å, Z: 40.0 Å. A higher exhaustiveness, set at 64, was used to achieve relatively high accuracy.

Supplementary figures

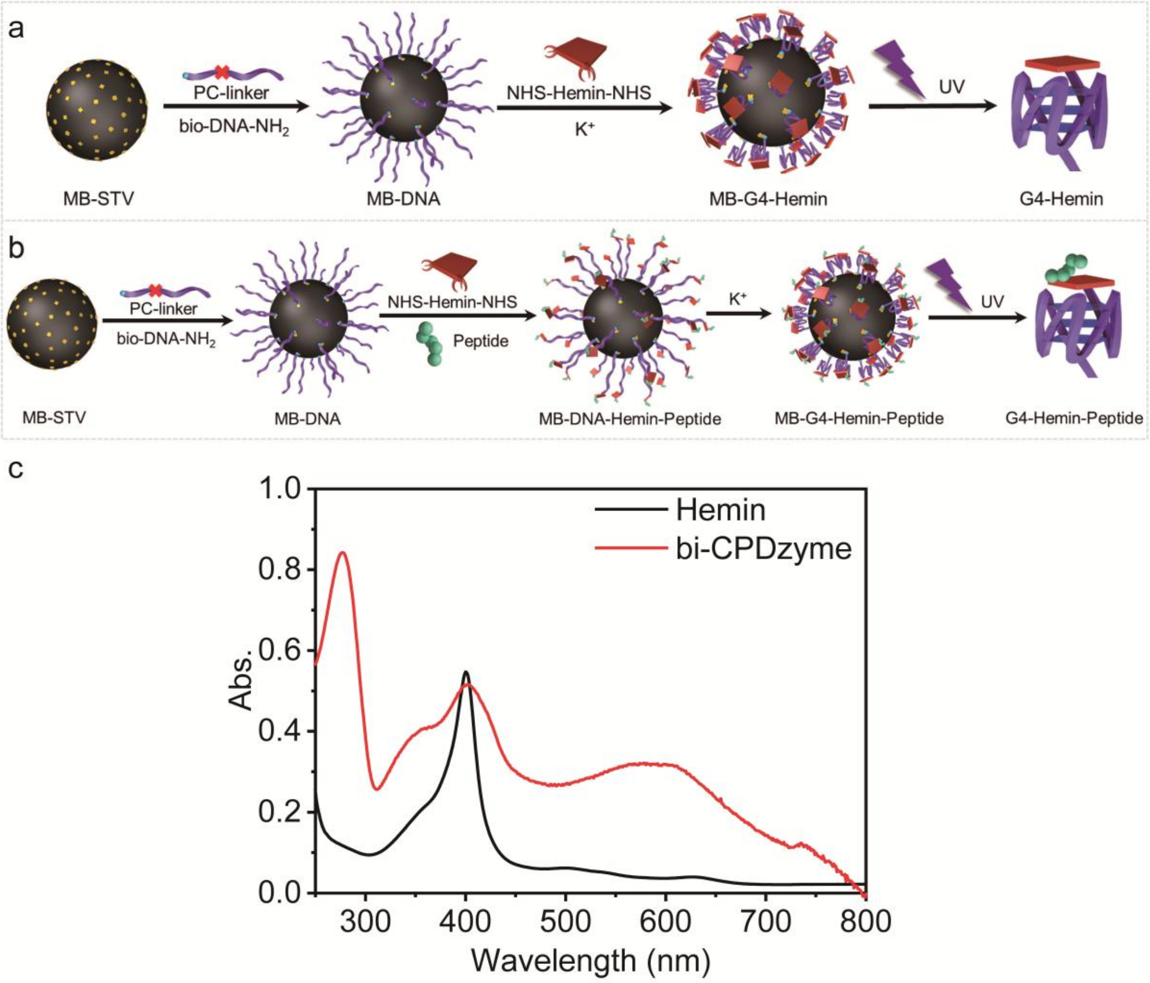

Figure S1. Schematic diagram of the preparation process of (a) G4-Hemin, (b) G4-Hemin-peptide. (c) UV absorption spectra of hemin and bi-CPDzyme.

The results in **Figure S1c** showed that bi-CPDzyme with characteristic DNA and hemin peaks around 260 nm and 404 nm, respectively.

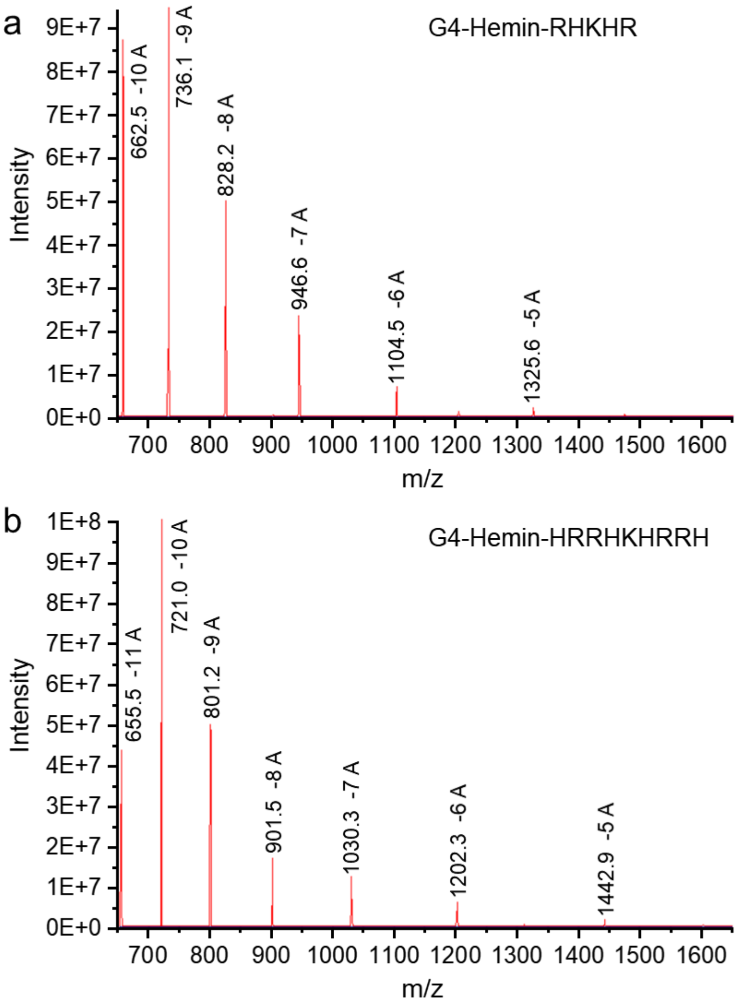

Figure S2. Mass spectra of bi-CPDzymes (a) G4-Hemin-RHKHR and (b) G4-Hemin-HRRHKHRRH.

The measured masses of two representative bi-CPDzymes, G4-Hemin-RHKHR and G4-Hemin-HRRHKHRRH, by ESI-MS are 6633 and 7219.5, respectively, which is in accordance with the calculated masses 6632.01 and 7218.67.

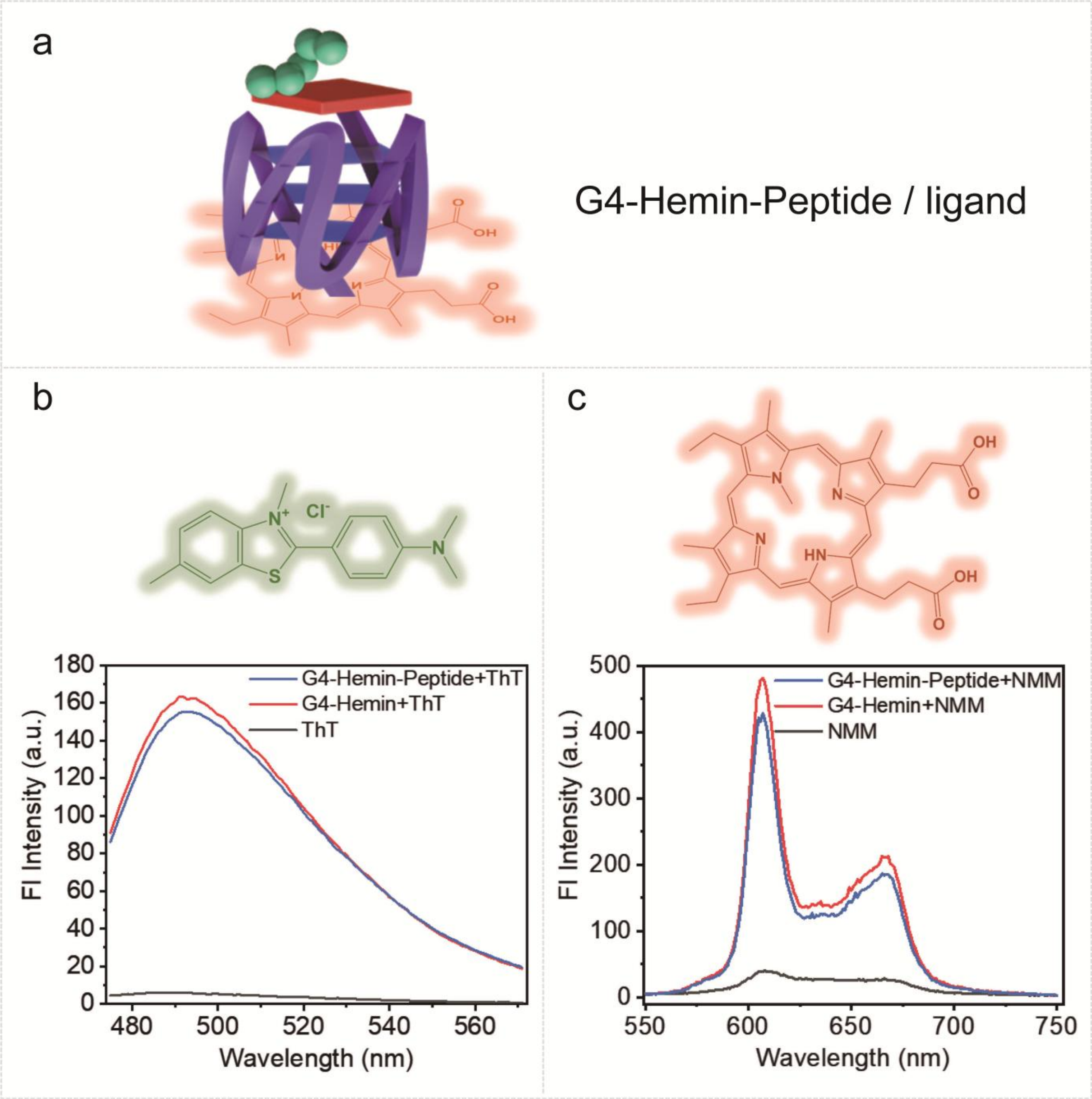

Figure S3. (a) Schematic diagram of fluorescent ligand interacts with G4-Hemin-peptide. Fluorescence spectra of (b) ThT and (c) NMM in the presence G4-Hemin or G4-Hemin-peptide.

The fluorescent ligands interact with G4-Hemin or G4-Hemin-peptide in the 5' plane *via* π-π stacking, evidencing the G4 formation in the bi-CPDzyme by the enhancement of ligands' fluorescent signal.

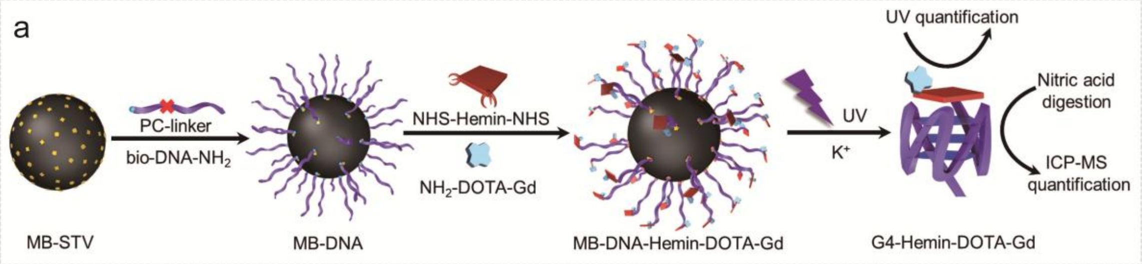

b

	156 Gd	157 Gd	158 Gd	160 Gd	Ave.(ppb)	Ave.(nM)
Sample 1	1.470	1.481	1.504	1.428	1.471±0.032	9.35±0.20
Sample 2	1.496	1.539	1.566	1.526	1.532±0.029	9.74±0.18

Figure S4. (a) Schematic diagram of the preparation process of G4-Hemin-DOTA-Gd, with the difference that the peptide in bi-CPDzyme was replaced by NH2-DOTA-Gd, and the two quantification methods, UV absorption and ICP-MS, for bi-CPDzyme. (b) Quantification results of G4-Hemin-DOTA-Gd by ICP-MS. The data shown in Panel B is very similar to the results collected by UV absorption. For instance, when the concentration of a given sample quantified by UV was 10 nM, the results measured by ICP-MS were 9.35 and 9.74 nM.

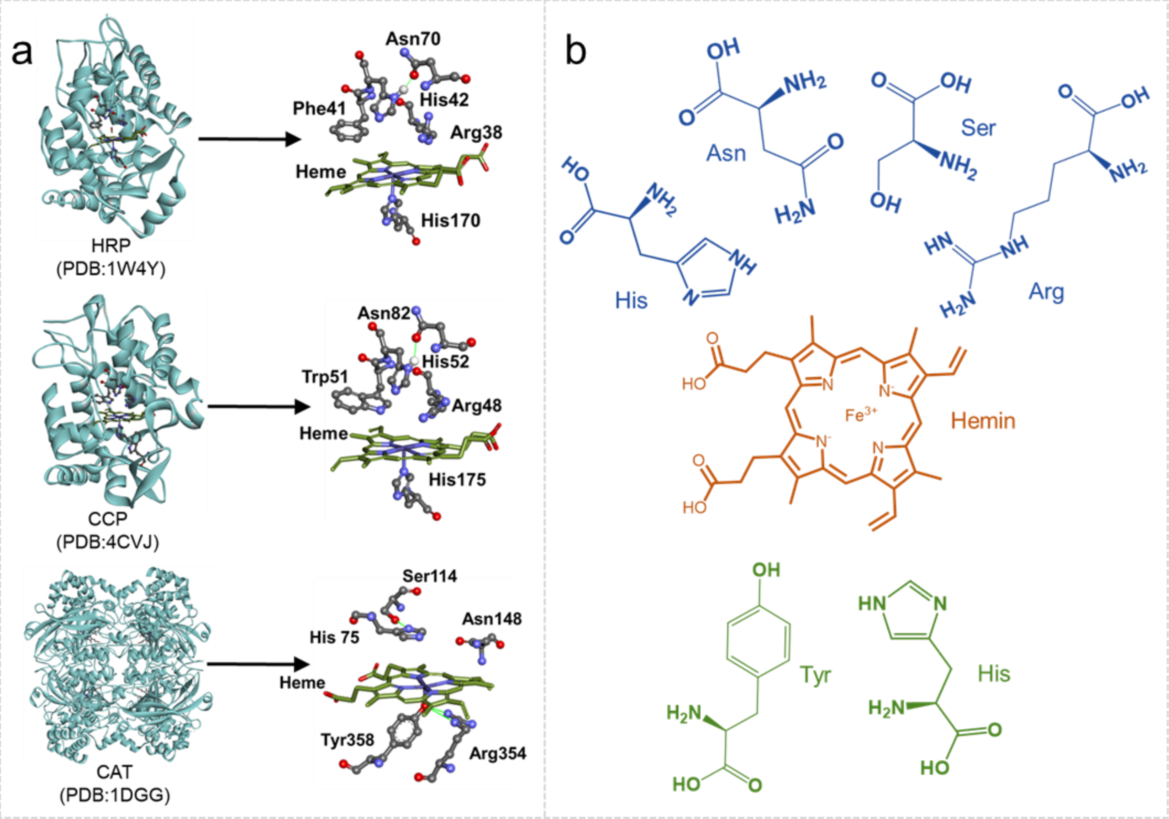

Figure S5. (a) Amino acid residues in the heme-binding region of HRP, CcP and CAT. (b) Key amino acid residues in the proximal and distal regions of the natural enzyme heme.

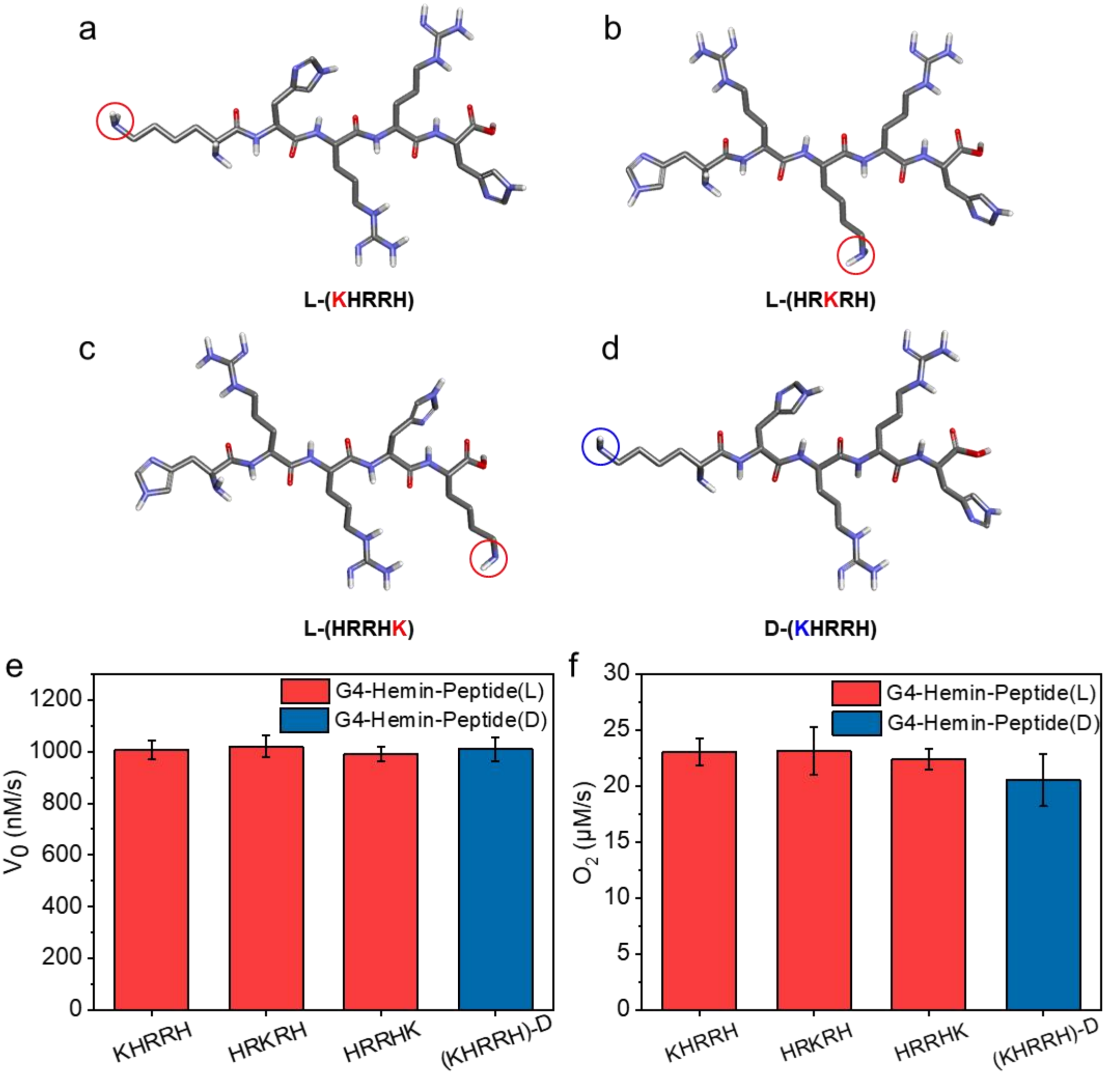

Figure S6. Schematic structure of Lys at the (a) N-terminus, (b) intermediate, (c) C-terminus of L-type peptides, and the (d) N-terminus of D-type peptides and the corresponding (e) POD and (f) CAT activities of bi-CPDzymes.

Taking the HRRH peptide as an example, when the Lys residue was located at the N-, C-terminal ends, and intermediate of the L-type peptide, the POD and CAT activities of bi-CPDzyme was almost equal, demonstrating bi-CPDzyme activities independent of Lys position. Interestingly, a similar situation is observed in D-type peptides. These results suggest that the linker at different spatial positions leads to changes in the spatial conformation of the peptide, but it does not affect the catalytic activity.

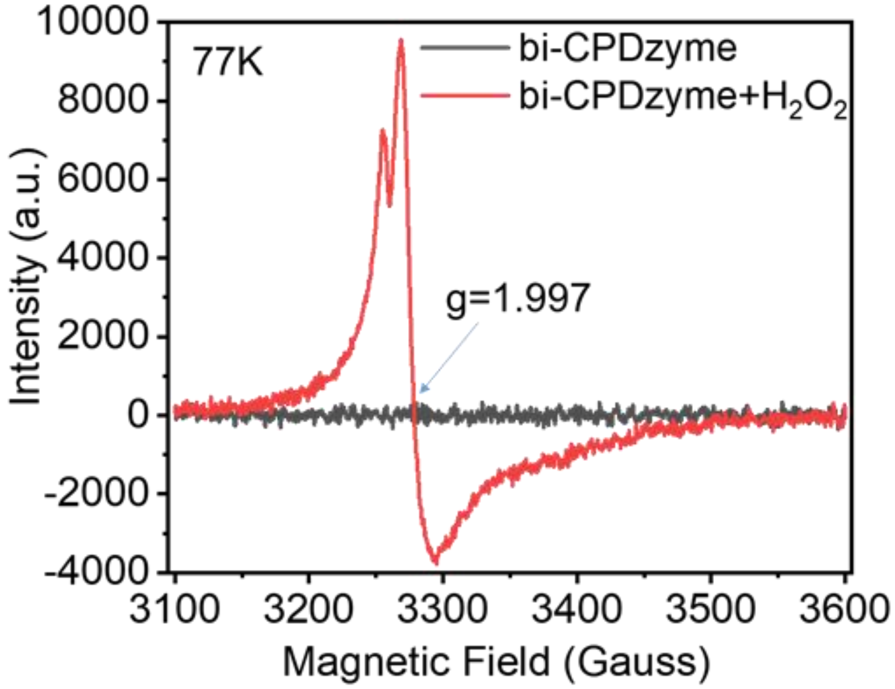

Figure S7. EPR spectra of bi-CPDzyme and bi-CPDzyme + H_2O_2 (77 K, microwave power 19.73 mW; microwave frequency 9.165 GHz, gain 2* 10^2, and modulation amplitude 1 G).

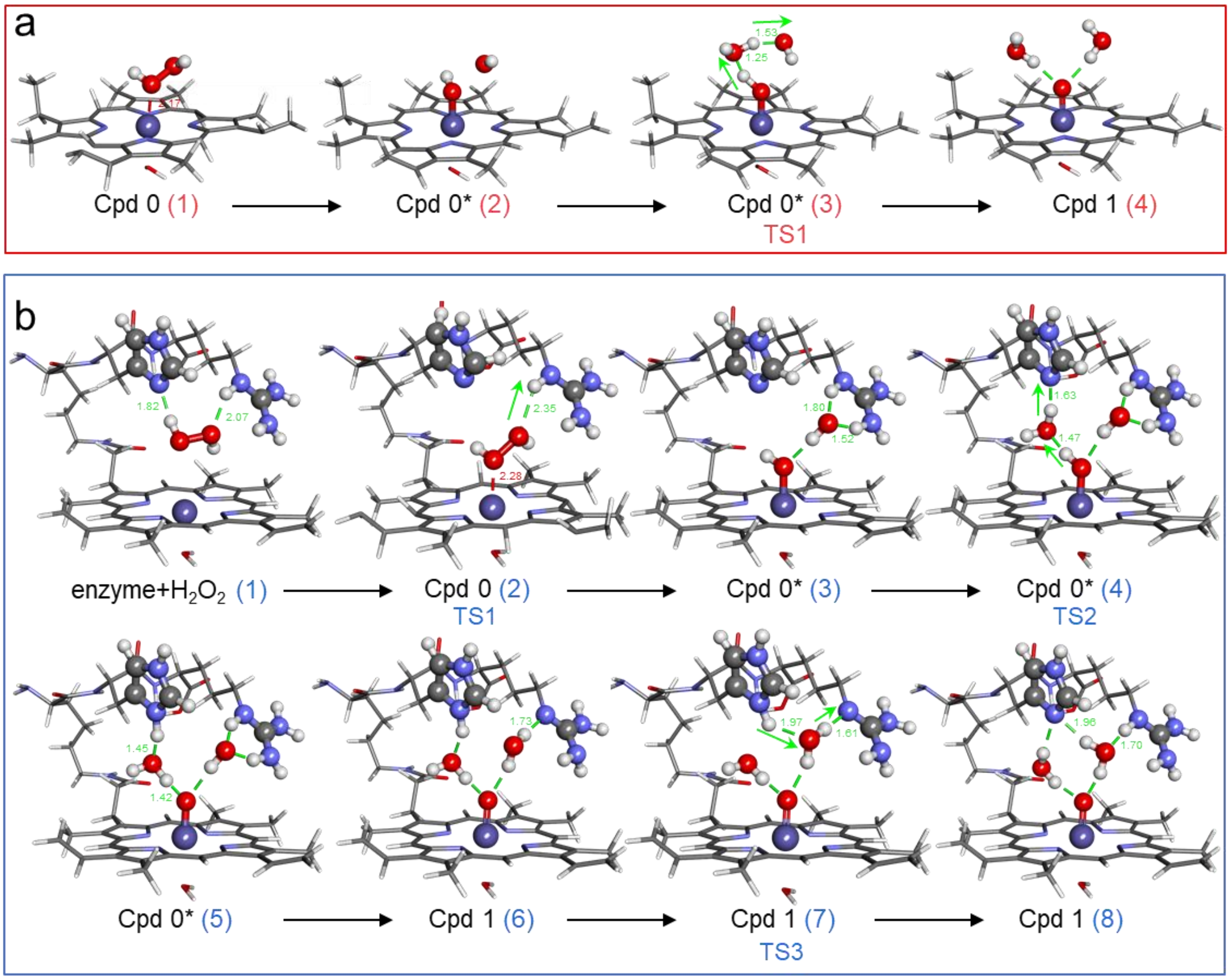

Figure S8. DFT derived configurations of the intermediates for H_2O_2 reacted with for (a) G4-Hemin and (b) G4-Hemin-KHR.

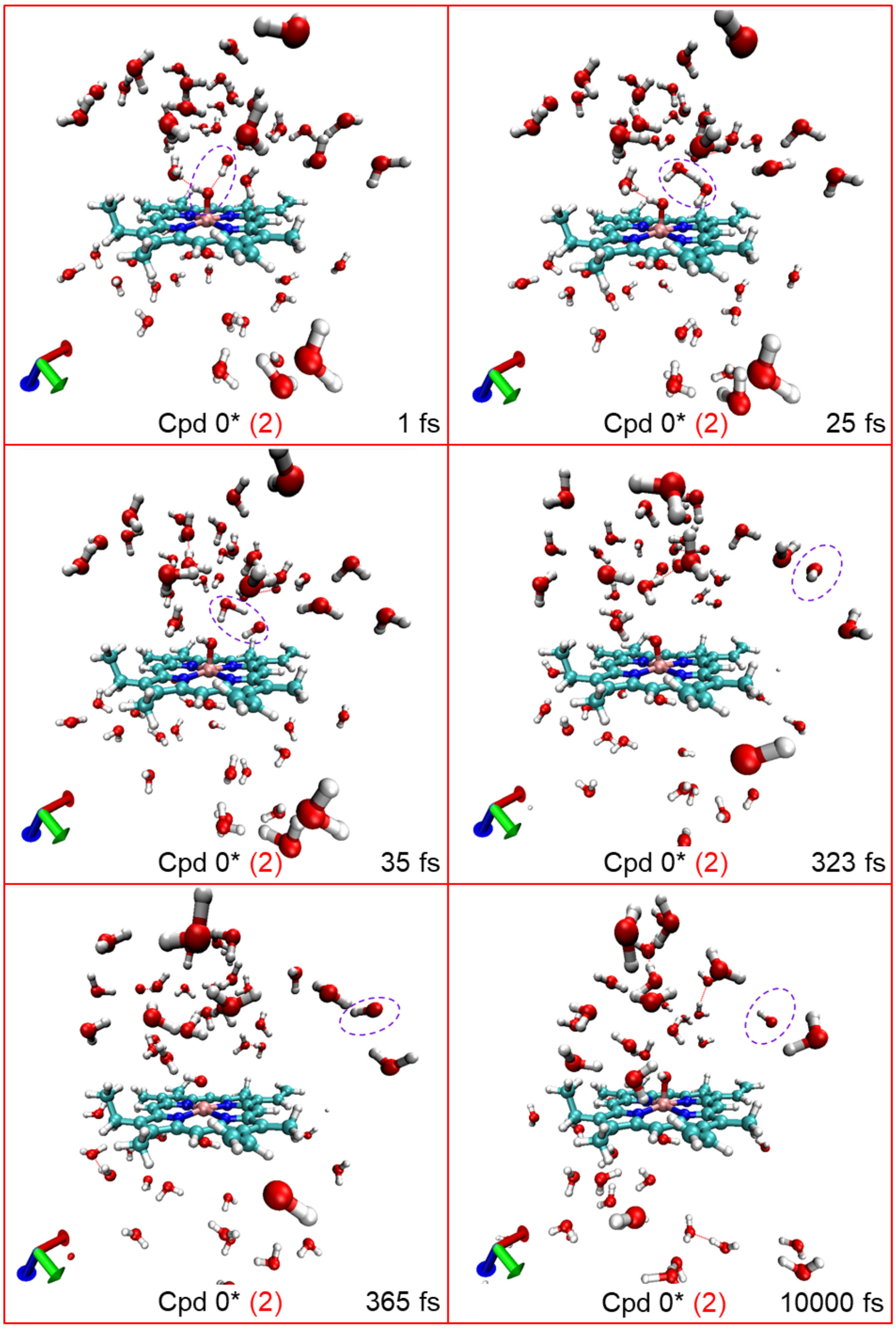

Figure S9. Snapshot series illustrate the evolution of the generated OH^- and Fe-OH group within the AIMD trajectory of the G4-Hemin system, with key changes highlighted by purple circles.

In the G4-Hemin system **(Movie S3)**, AIMD simulations spanning 10,000 fs revealed that the O-H bond distance in the Fe-OH moiety of Cpd0* exhibited minimal fluctuations (approximately 1 Å), indicating its remarkable stability. The OH^-, generated from H_2O_2 reaction with the iron center, first engaged in brief interactions with adjacent water molecules before diffusing away from the Fe-OH site. Crucially, the proton in Cpd0* persisted due to inefficient abstraction pathways, thus hindering formation of the high-valent iron-oxo species Cpd I. This kinetic bottleneck suggests that the need to modulate the proton transfer network, facilitating downstream catalytic steps.

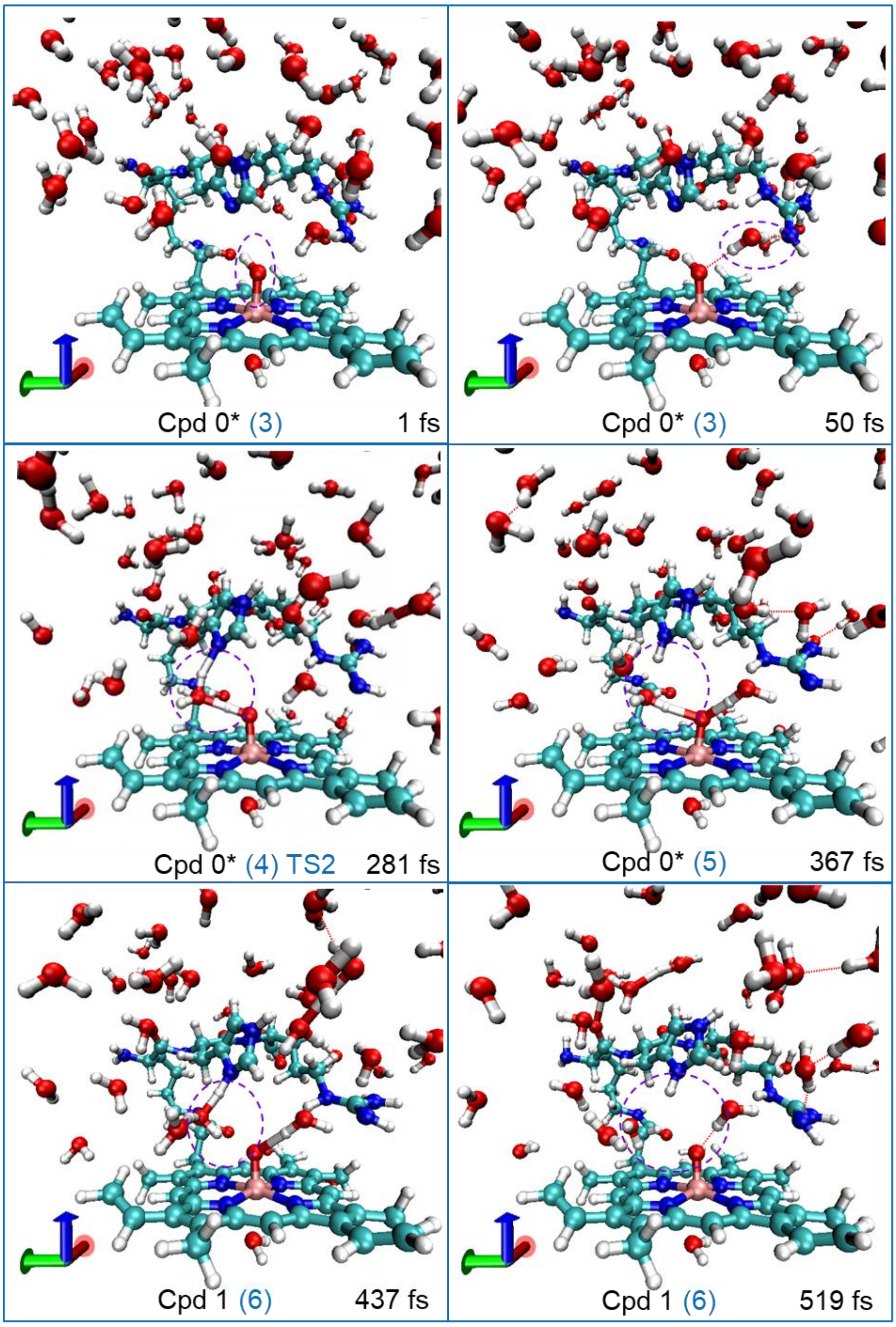

Figure S10. Snapshots provide a direct visualization of the Cpd0*→Cpd I transition captured during the AIMD trajectory in the G4-Hemin-KHR system; the key changes are highlighted by purple circles in the figure.

In the G4-hemin-KHR system **(Movie S4)**, AIMD simulations indicate that only 437 fs required for Cpd0* to evolve into Cpd I. At 281 fs, the His-N proton-acceptor establishes a water-mediated hydrogen-bond network with Fe-OH, matching the transition state identified in DFT as Cpd0*(4)–TS2. At 367 fs, the water proton is captured by the histidine, and within the next 70 fs a second proton is abstracted from the Fe-OH, yielding a stable water molecule and the high-valent iron-oxo species Cpd I. These kinetic simulations demonstrate that the amino-acid residue, functioning as an indispensable proton shuttle, removes the kinetic bottleneck observed in the G4-Hemin system when such a proton-transfer network is absent.

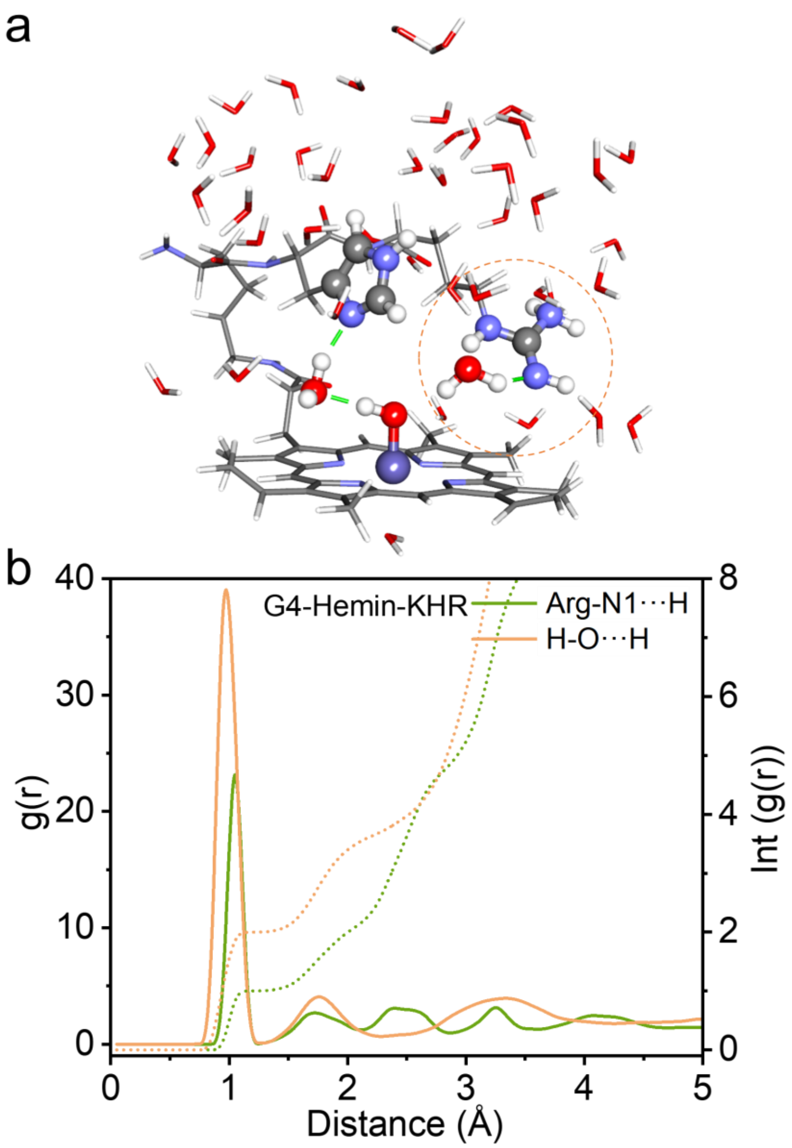

Figure S11. (a) Snapshot for Arg-N confining the generated free OH^- from the cleavage of H_2O_2 in G4-Hemin-KHR system. Arg-N1 ··H ··O-H in the red circle corresponds to the panel b. (b) The pair distribution functions of the Arg-N and the O of free OH^- to H atoms.

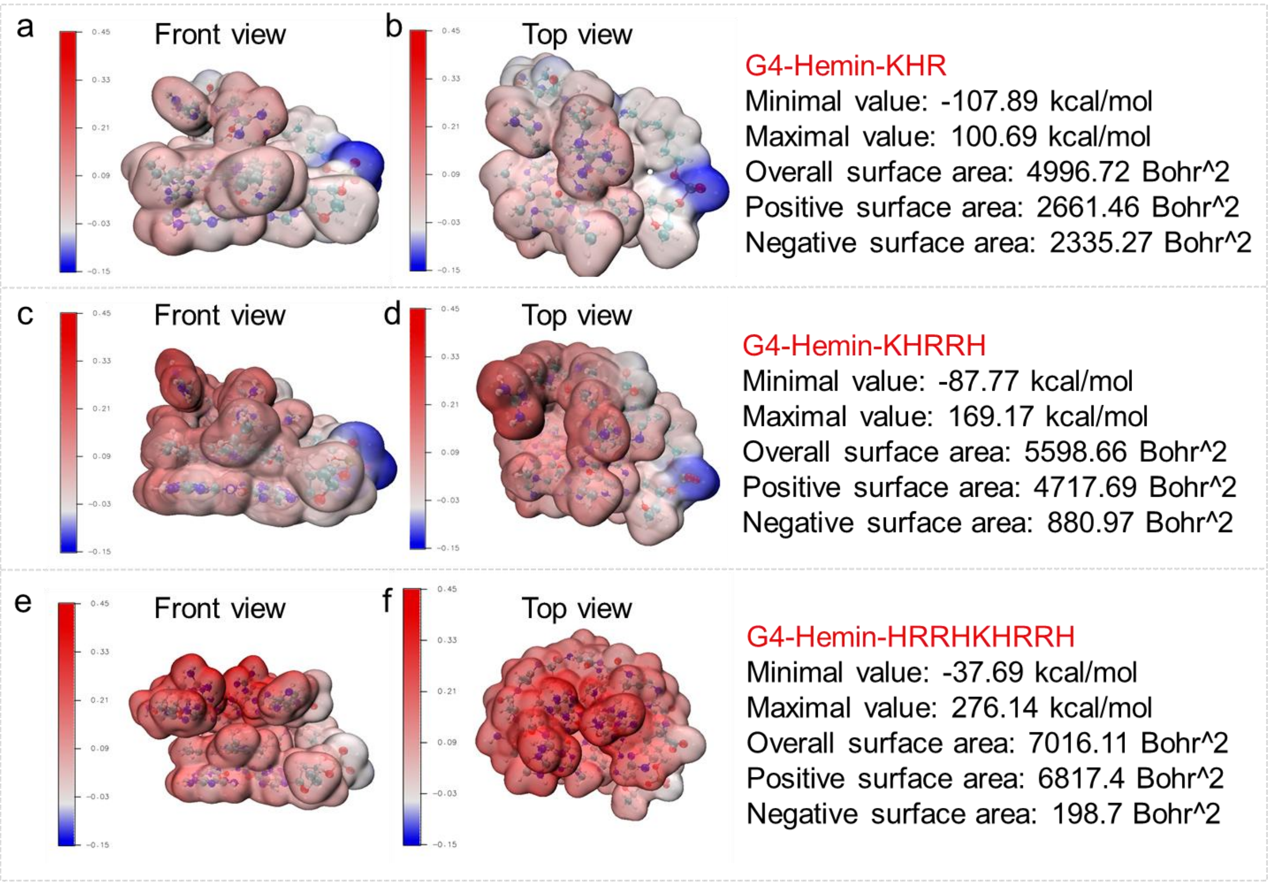

Figure S12. Electrostatic potential (ESP) mapped molecular van der Waals surface of bi-CPDzyme. The unit is in kcal/mol. Locally positively and negatively charged surfaces of ESP are represented as red and blue, respectively.

Electrostatic potential (ESP) mapping of the van der Waals surface of the bi-CPDzyme shows an increase in the maximal value from 100.69 to 276.14 kcal/mol as well as an increase in the positive surface area share from 53.3% to 97.2% with an increase in the number of H+R pairs. The possible reason is that the pK_a of guanidine group on Arg is 12.48, which is positively charged under neutral conditions.

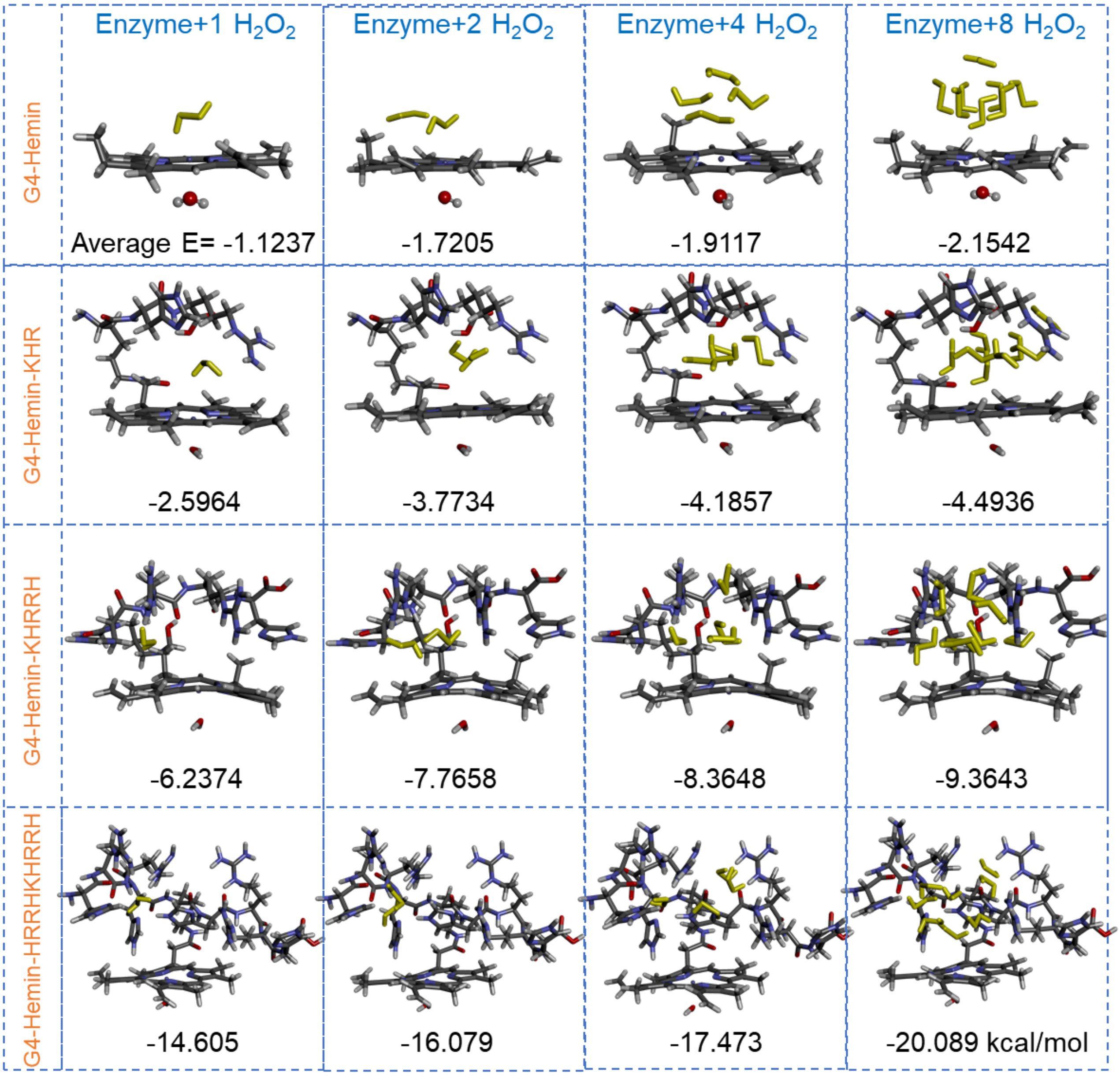

Figure S13. Docking binding models and average binding energies of G4-Hemin and bi-CPDzymes with one, two, four, and eight H_2O_2.

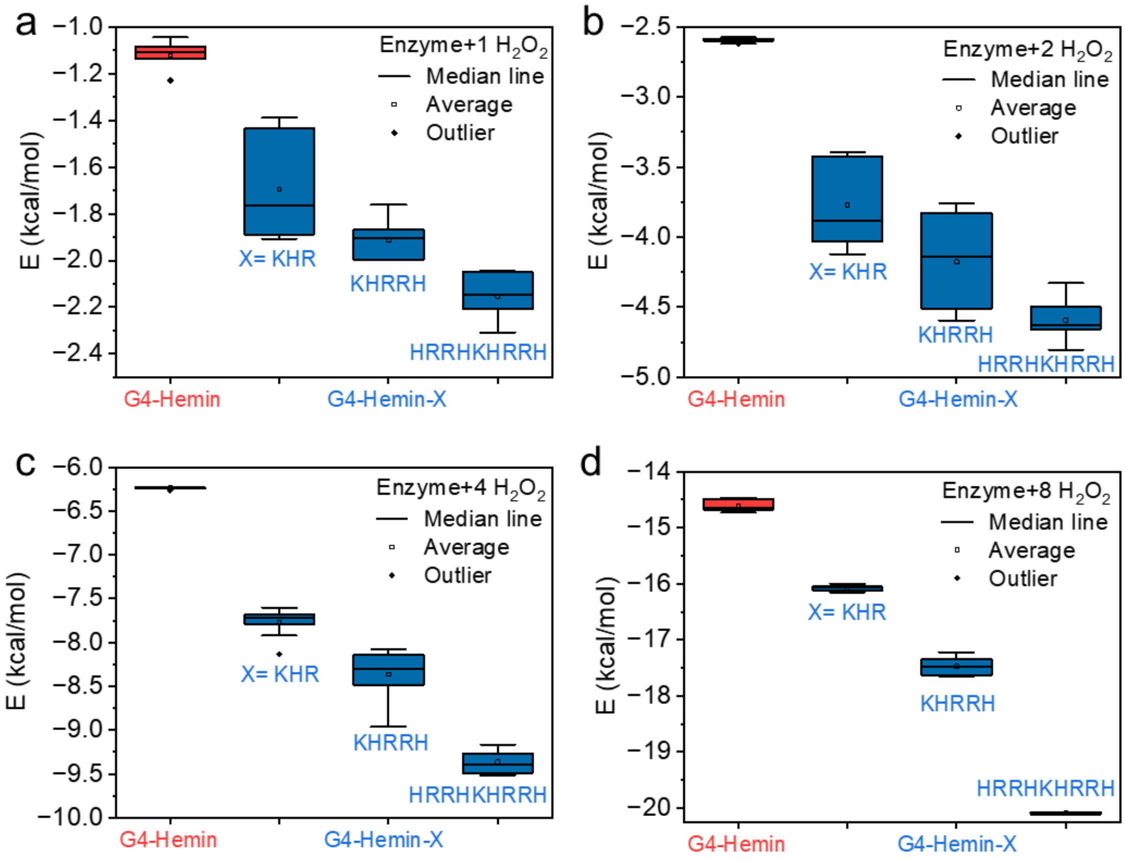

Figure S14. Statistical plot of docking binding energies of G4-Hemin and bi-CPDzymes with one, two, four, and eight H_2O_2.

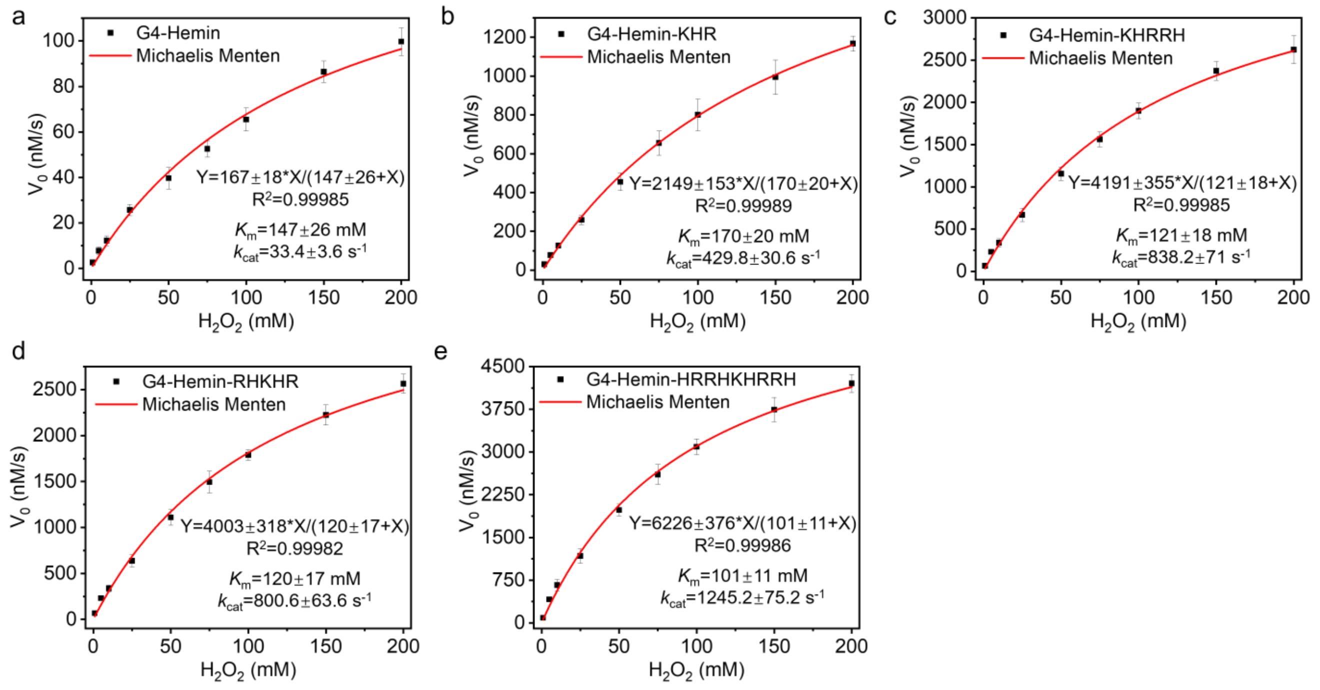

Figure S15. POD activity fitting curve based on the Michaelis-Menten model of bi-CPDzyme from the steady-state kinetics by varying the substrate concentration. POD experiments were performed in 10 mM Tris−HCl buffer (pH 7, 100 mM K^+) containing 5 nM bi-CPDzymes and 5 mM ABTS.

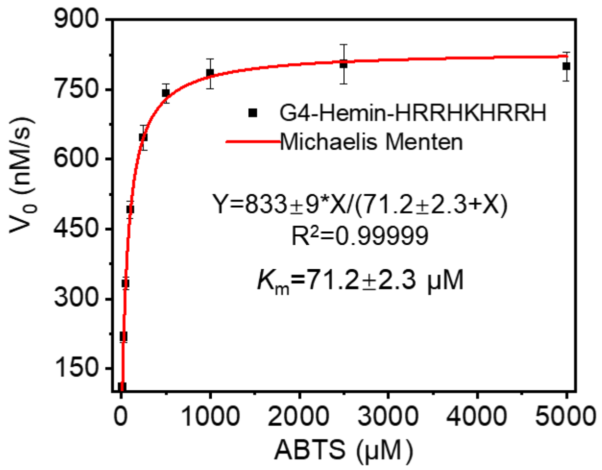

Figure S16. The saturation curve corresponding to the ABTS oxidation by G4-Hemin-HRRHKHRRH at different concentrations of ABTS. Experiments were performed in 10 mM Tris-HCl buffer (pH 7, 100 mM K^+) containing 15 nM bi-CPDzyme and 5 mM H_2O_2.

As shown in **Figure S16**, K_m^{ABTS} = 71 μM. In **Figure S15**, we measured the POD kinetic parameters (H_2O_2) of bi-CPDzyme at 5 mM ABTS. Thus, the POD kinetic parameters is a zero-order reaction with the substrate ABTS, that is independent of the concentration of ABTS.

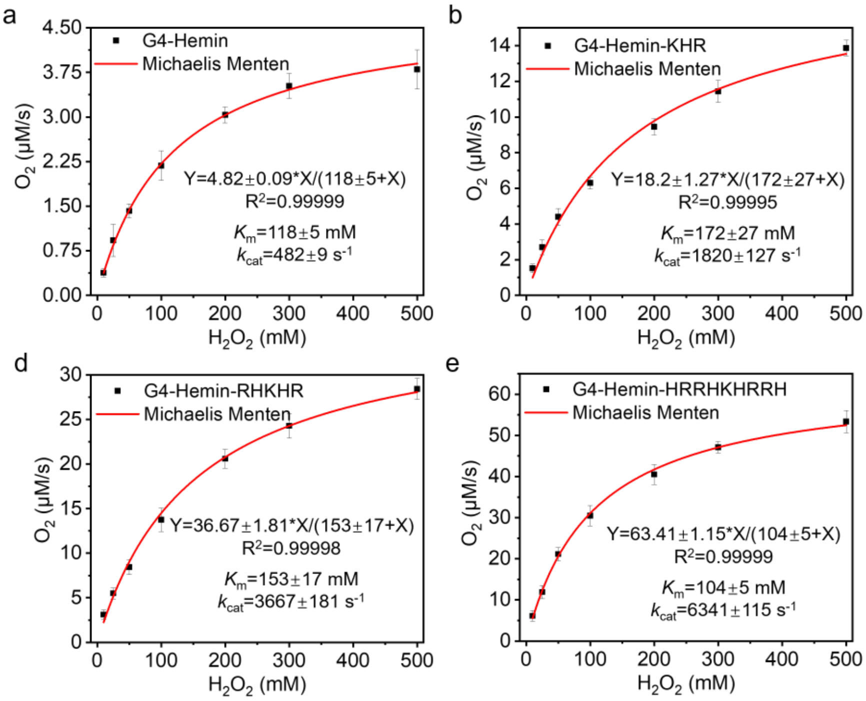

Figure S17. CAT activity fitting curve based on the Michaelis-Menten model of bi-CPDzyme from the steady-state kinetics by varying the substrate concentration. CAT experiments were performed in 10 mM Tris-HCl buffer (pH 7, 100 mM K^+) containing 10 nM bi-CPDzymes.

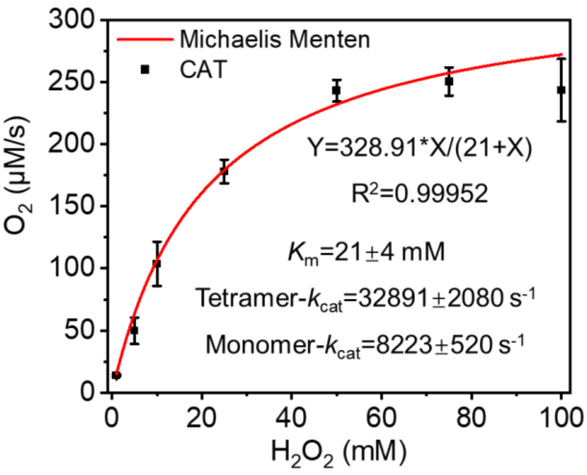

Figure S18. Michaelis−Menten fitting curve corresponding to the oxidation rate of CAT at variable concentrations of H_2O_2. Experiments were performed in 10 mM Tris-HCl buffer (pH 7, 100 mM K^+) containing 10 nM CAT.

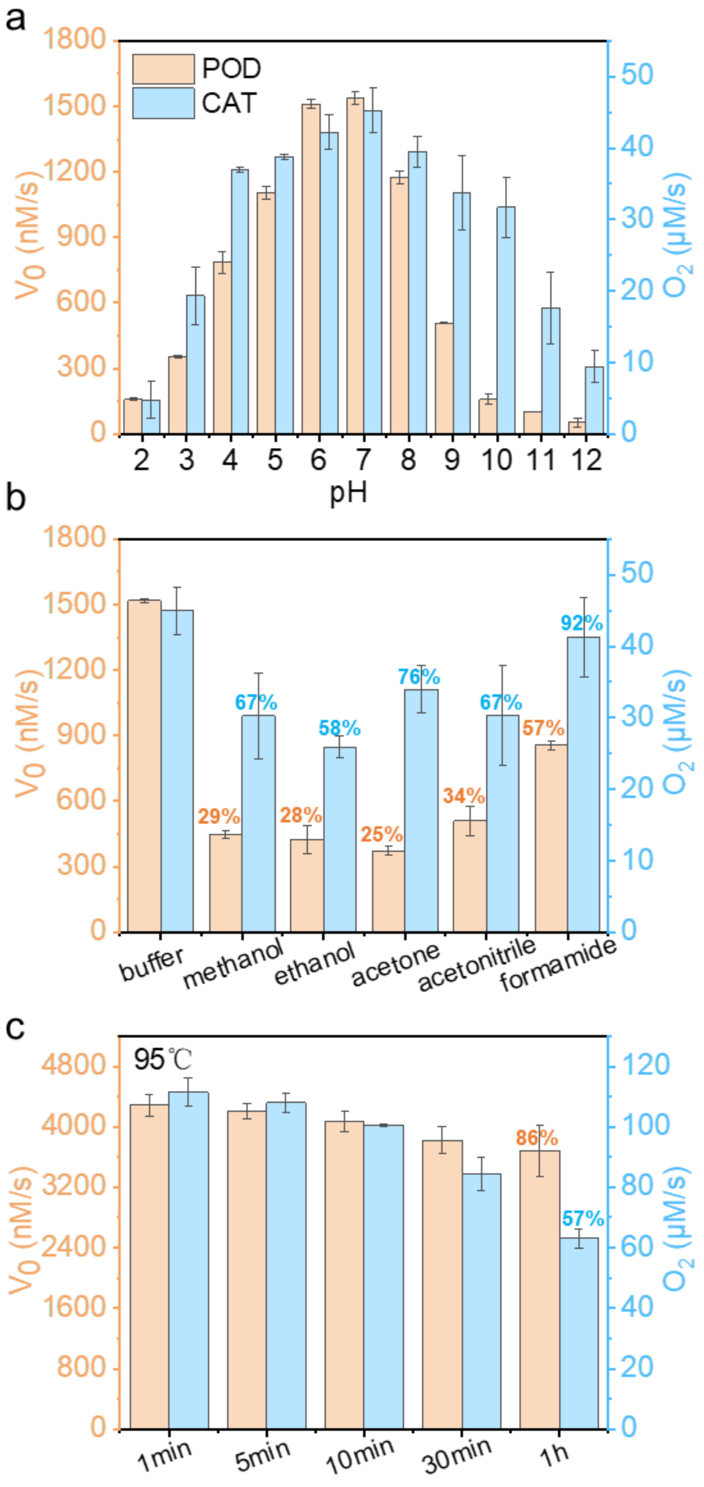

Figure S19. Chemical robustness of G4-Hemin-HRRHKHRRH. (a) POD and CAT catalytic activity in the presence of G4-Hemin-HRRHKHRRH for different pH at 25 ℃; (b) in conventional aqueous buffer and different organic-water mixed solutions (50%, v/v); (c) at 95 ℃ for different time. Orange and blue color represent POD and CAT activity, respectively.

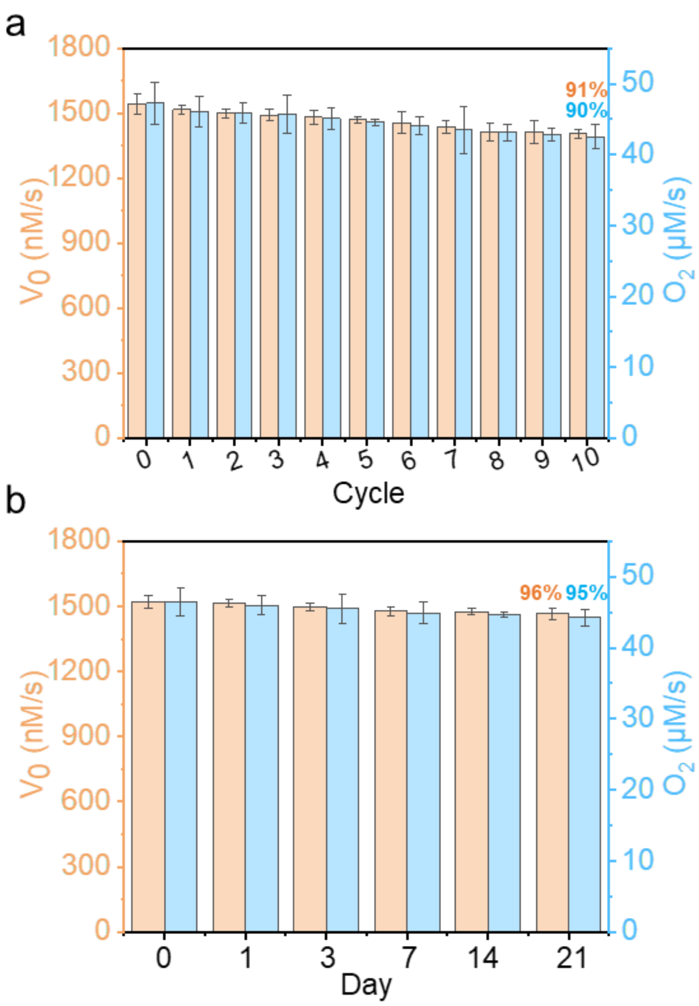

Figure S20. (a) Reusability of MB-G4-Hemin-HRRHKHRRH over 10 consecutive cycles. (b) Long-term stability of MB-G4-Hemin-HRRHKHRRH stored at room temperature for 21 days; The activity is expressed as a percentage of the initial value. Orange and blue color represent POD and CAT activity, respectively.

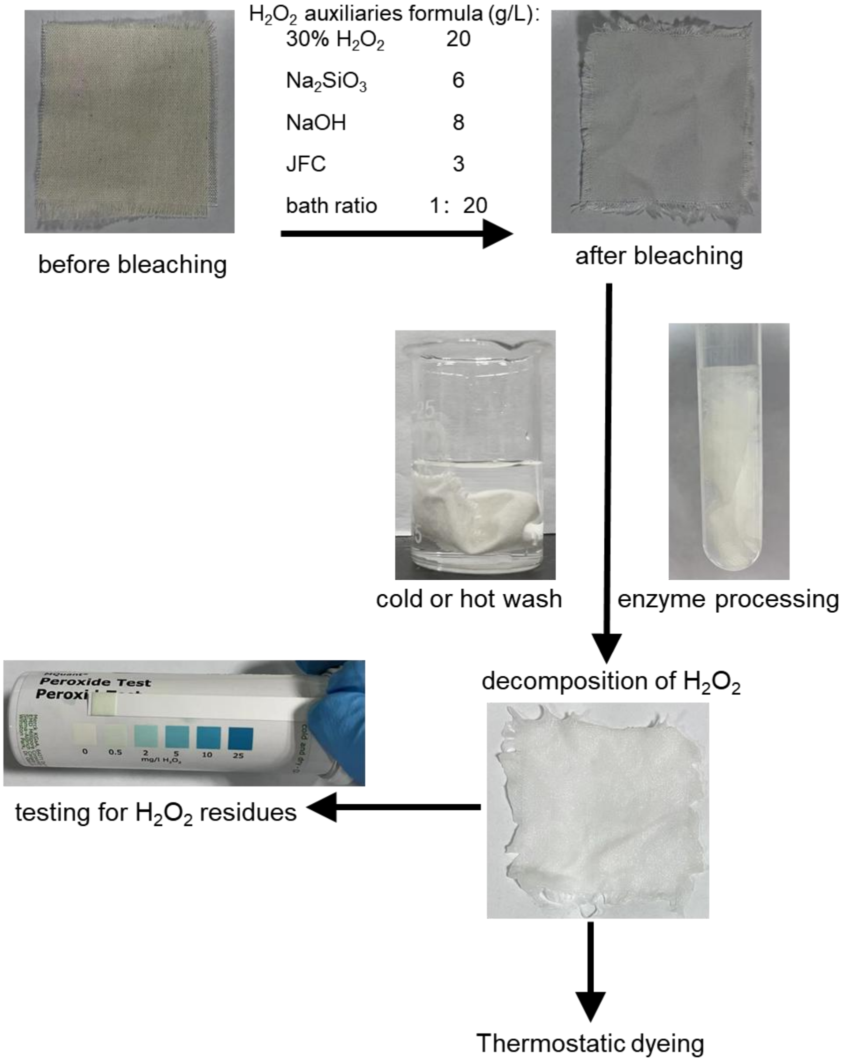

Figure S21. The bleaching post-treatment process and formulation of bleach solution.

A piece of fabric was bleached at high temperature with an H_2O_2 auxiliaries (30% H_2O_2, Na_2SiO_3, NaOH, JFC; bath ratio 1:20) for 30 minutes, then the excess H_2O_2 was removed by washing or enzyme/bi-CPDzyme treatment, and then the residual H_2O_2 was quantified with an H_2O_2 test paper, and finally the staining was carried out at 60 ^{o}C.

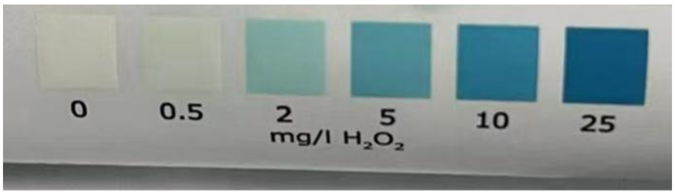

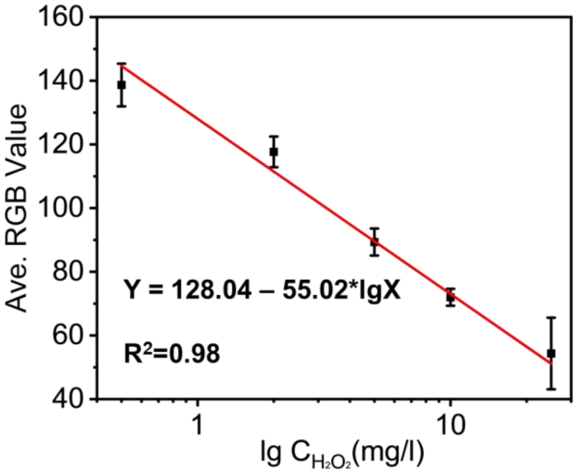

Figure S22. Photograph of colorimetric card (above) and plotted standard curve (below).

RGB values were read from the colorimetric card photograph and a standard curve was plotted Y=128.04-55*lgX, R^2=0.98. A cell phone was used to take a picture of the H_2O_2 strips in order to identify the RGB values in the photograph, and the residual H_2O_2 was quantified by the standard curve.

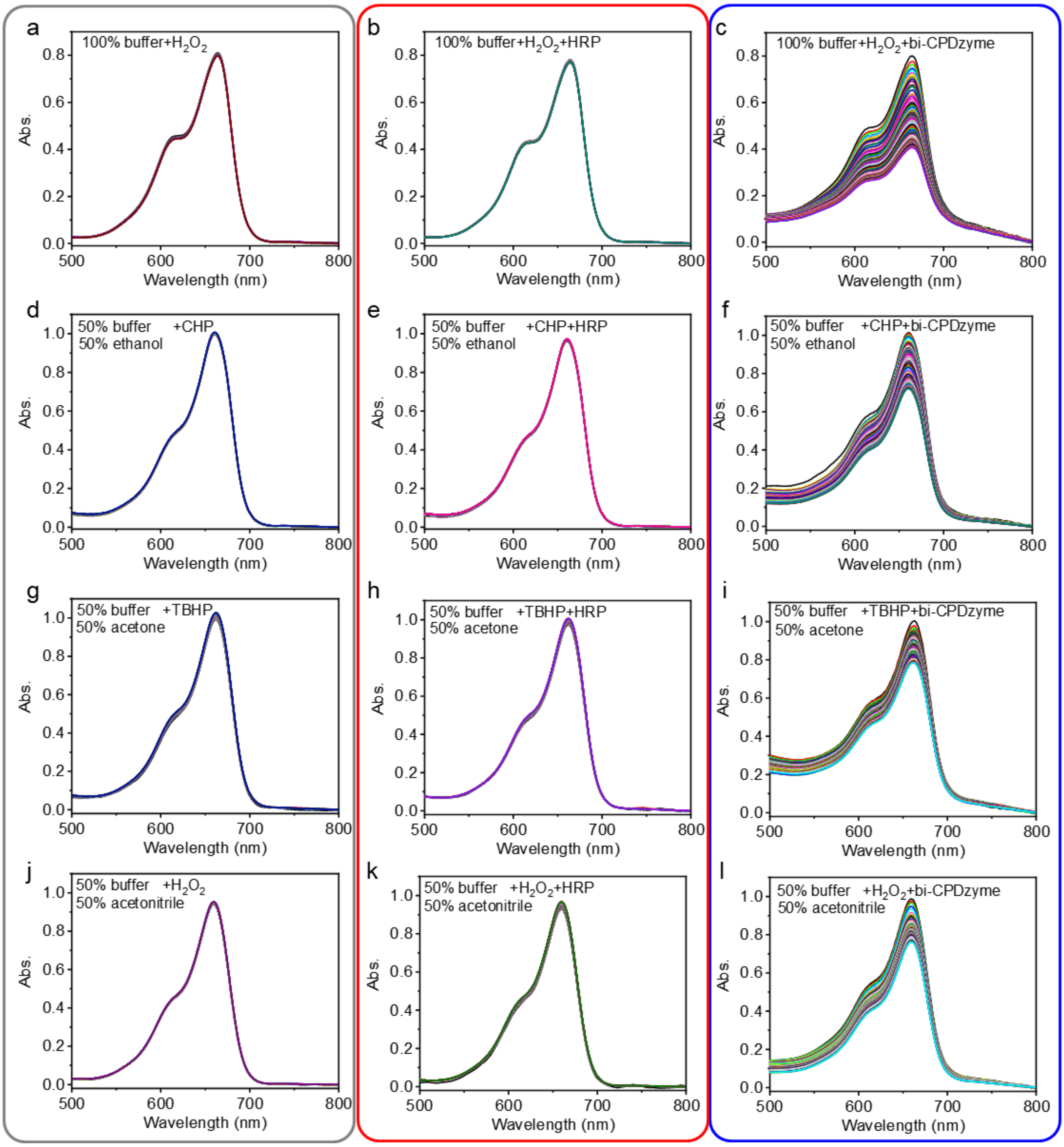

Figure S23. The UV spectra of the degradation BB9 of background (gray frame), HRP (red frame) and bi-CPDzyme (G4-Hemin-HRRHKHRRH) (blue frame) under various conditions. (a-c) Degradation of BB9 under conventional buffer conditions with H_2O_2 as oxidant; (d-f) Degradation of BB9 under 50% ethanol (v/v) with CHP as oxidant. (g-i) Degradation of BB9 under 50% acetone (v/v) with TBHP as oxidant. (j-l) Degradation of BB9 in 50% acetonitrile (v/v) with H_2O_2 as oxidant.

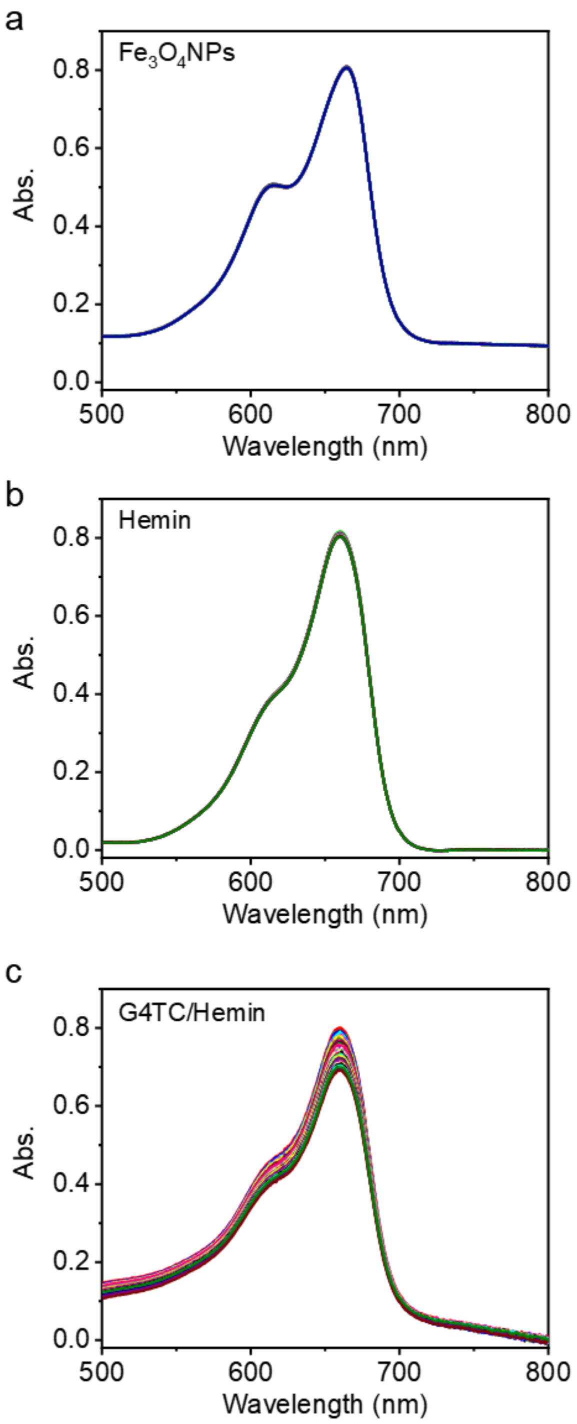

Figure S24. The UV spectra of the degradation of BB9 by (a) Fe_3O_4 NPs, (b) Hemin and (c) G4TC/Hemin under conventional buffer conditions with H_2O_2 as oxidant. Fe_3O_4 NPs [10] was quantified using the molar concentration [11].

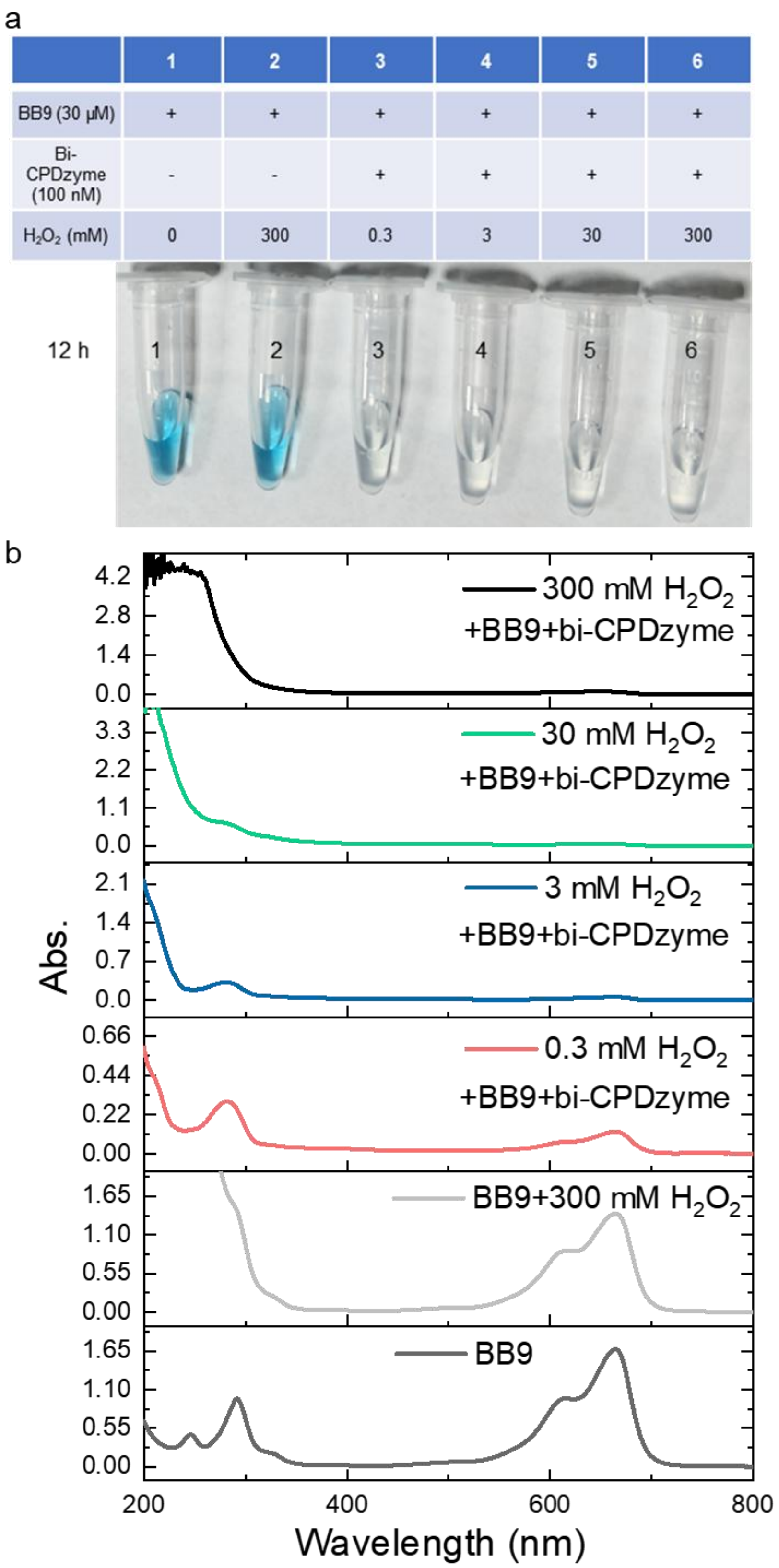

Figure S25. (a) Photographs of 30 μM BB9 treatment by 100 nM bi-CPDzyme for 12 h with different concentrations of H_2O_2 (0.3-300 mM). The control samples were conducted in the absence of bi-CPDzyme. (b) UV absorption spectra of corresponding samples in Panel A. Note, the samples were diluted in half for UV experiments here.

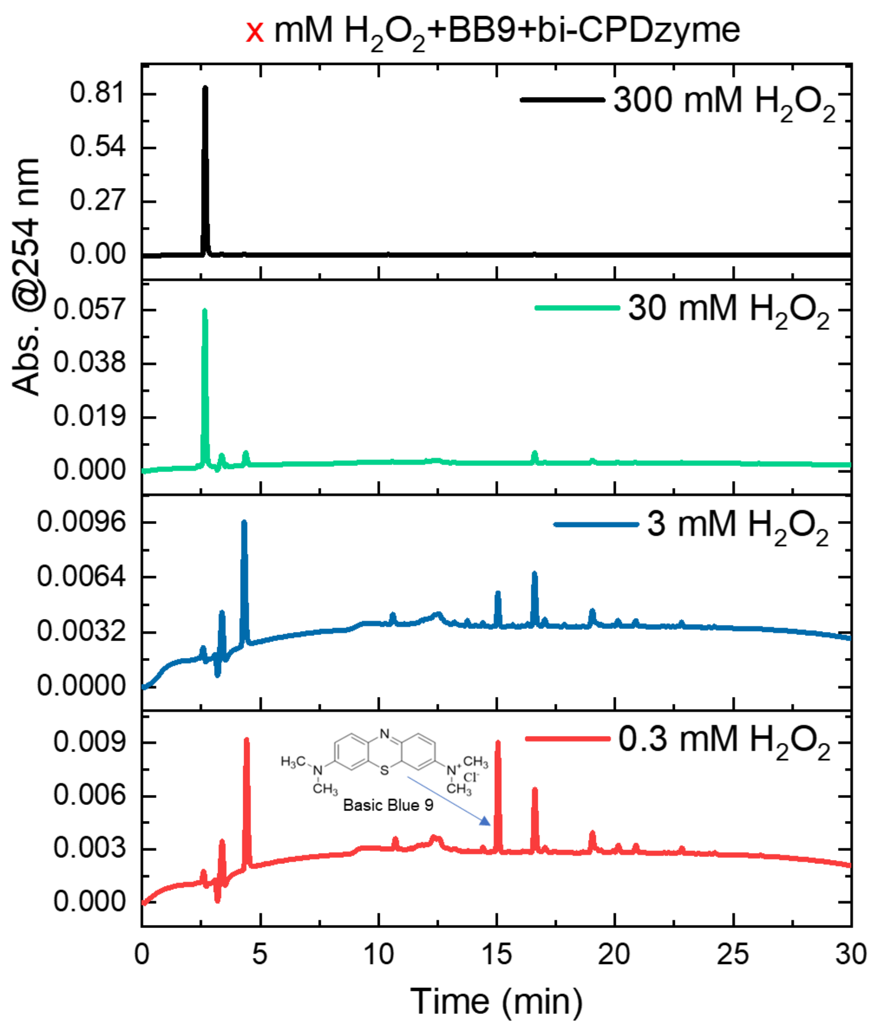

Figure S26. High performance liquid chromatography (HPLC) of BB9 after 12 h of 100 nM bi-CPDzyme treatment with different concentrations of H_2O_2. The UV wavelength is at 254 nm, and the mobile phase is acetonitrile and water. The peak appears around 15 min is BB9, and the inset shows the structural formula of BB9.

The HPLC data showed that the BB9 peak as well as the degradation product peak of BB9 gradually disappeared with the increase of H_2O_2 concentration, indicating that the high concentration of H_2O_2 could degrade BB9 into further products.

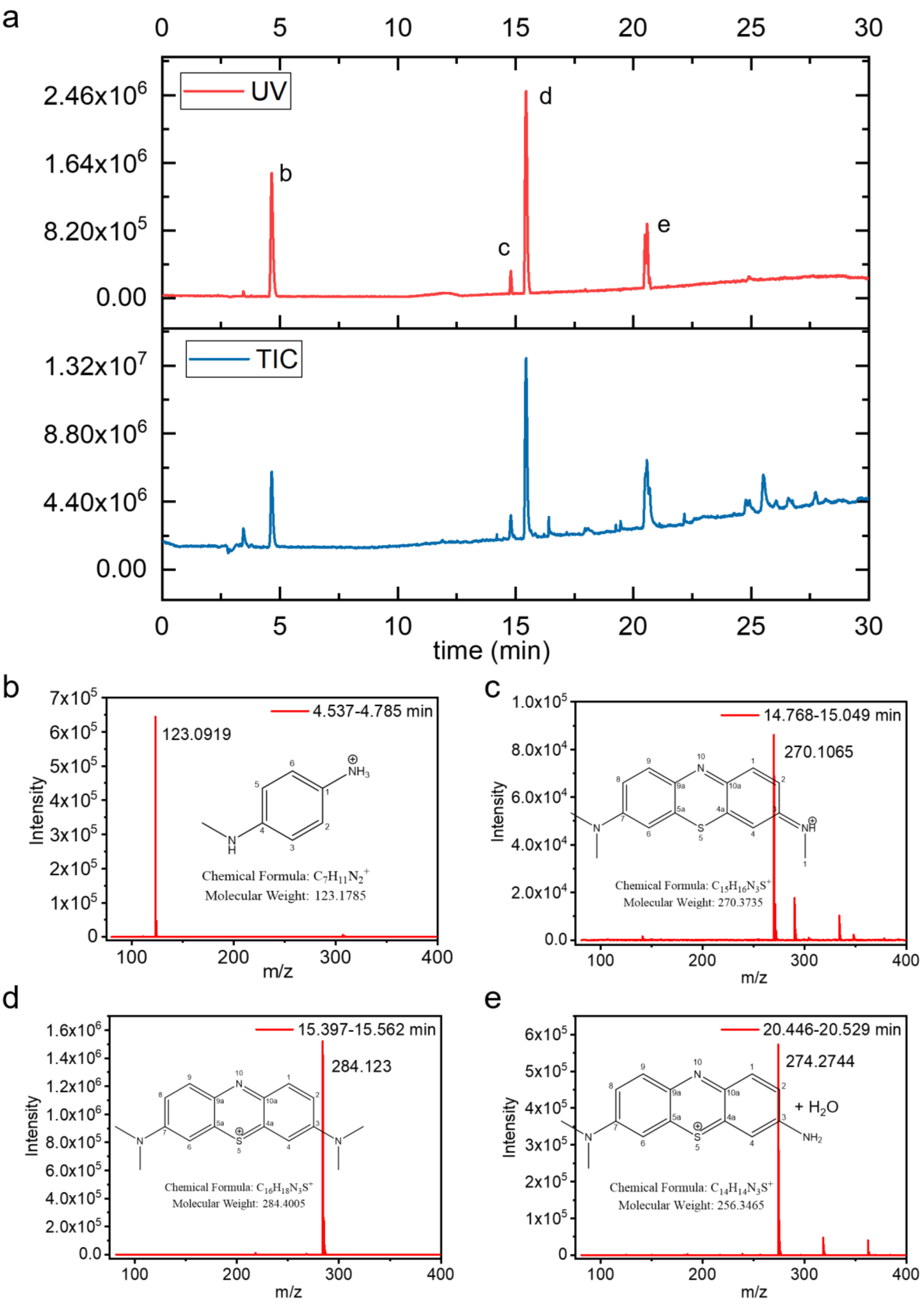

Figure S27. (a) Liquid chromatography-mass spectrometry (LC-MS) of BB9 after 12 h of treatment with bi-CPDzyme. The four peaks were interpreted in corresponding four Panels below. (b-e) Mass spectral peaks at (b) 4.54-4.79 min, M = 123.09, possibly $C_7H_{11}N_2^+$ (c) 14.77-15.05 min, M = 270.11, possibly $C_{15}H_{16}N_3S^+$ (d) 15.40-15.56 min, M = 284.12, possibly $C_{16}H_{18}N_3S^+$ and (e) 20.45-20.53 min, M = 274.27, possibly $C_{14}H_{14}N_3S^+$ plus one molecule of H_2O.

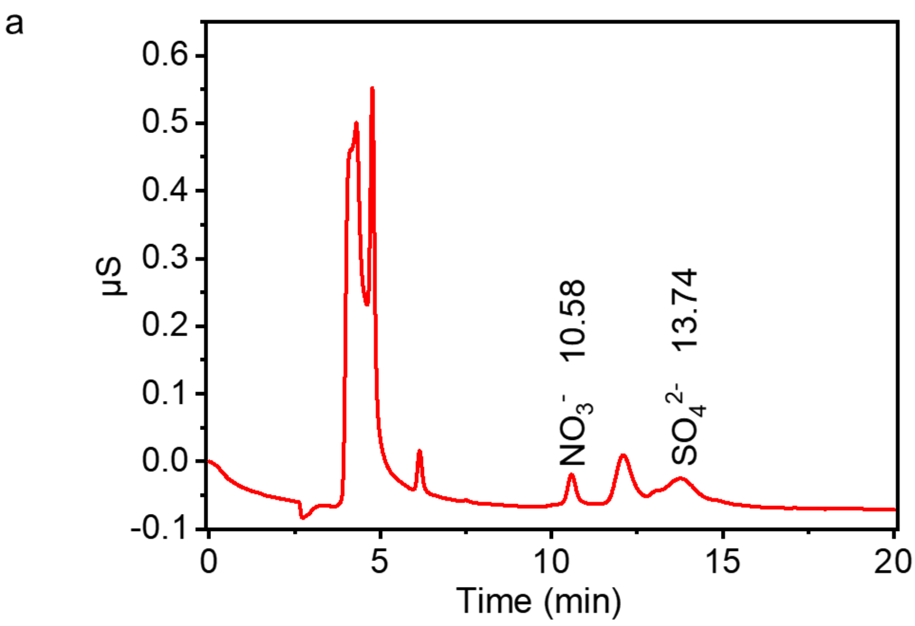

b

number	time	peak name	peak type	peak area	peak height	concentration (mg/L)
**1**	10.58	NO_3^-	BMB*	0.013	0.043	0.2323
**2**	13.74	SO_4^{2-}	BMB*	0.016	0.022	0.2697

Figure S28. (a) Ion chromatographic (IC) peaks of the terminal products NO_3^- and SO_4^{2-} of BB9. (b) The detail information of peak time, peak area, peak height and calculated concentrations about NO_3^- and SO_4^{2-} in Panel a. Experimental conditions: elution solution KOH (20 mM), flow rate 1 ml/min.

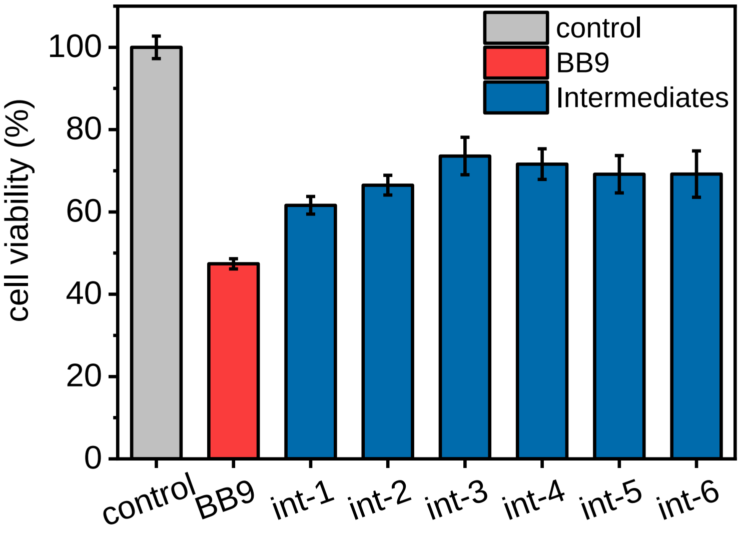

Figure S29. The cytotoxicity of BB9 and its degradation intermediates. The intermediates 1 to 6 corresponding to $C_{15}H_{16}N_3S^+$, $C_{14}H_{14}N_3S^+$, $C_{13}H_{12}N_3S^+$, $C_6H_8NS^+$, $C_7H_{11}N_2^+$, and $C_6H_9N_2^+$, respectively.

a

Location	Average Daily Flow (m^3/day)	Main Processes and Products
Tongxiang city, Zhejiang province, China	5000	Weaving, dyeing, printing of knitted fabric

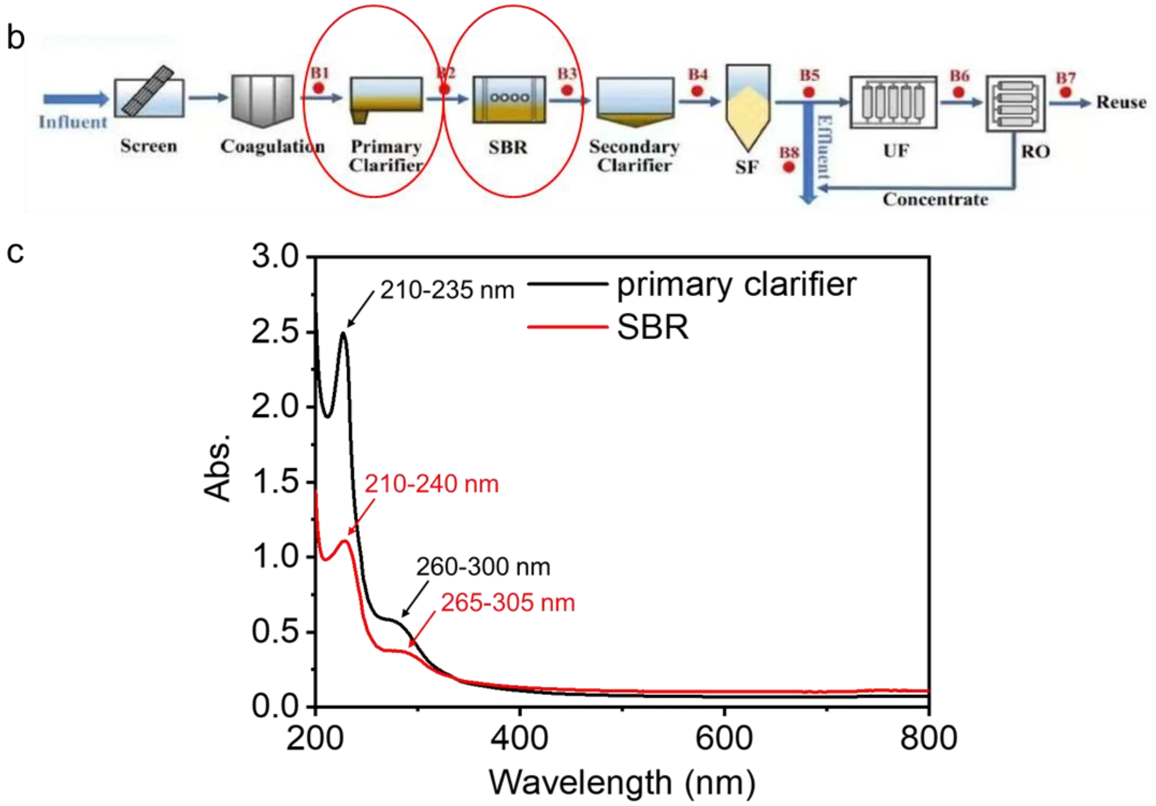

Figure S30. (a) Characteristics of textile dyeing wastewater treatment plant. (b) Water treatment processes of the textile printing and dyeing wastewater treatment plant. The red circles represent the sampling locations [12]. (c) UV absorption spectra of primary clarifier and SBR wastewater.

The UV absorption spectra were used to estimate the possible components of these real samples. **Figure S30c** shows that the Konjugierte at 210-240 nm may contain some conjugated dienes, aromatic aldehydes and ketones, and chromophore-substituted benzenes, and at 260-305 nm, some benzene rings or heterocyclic aromatic hydrocarbons.

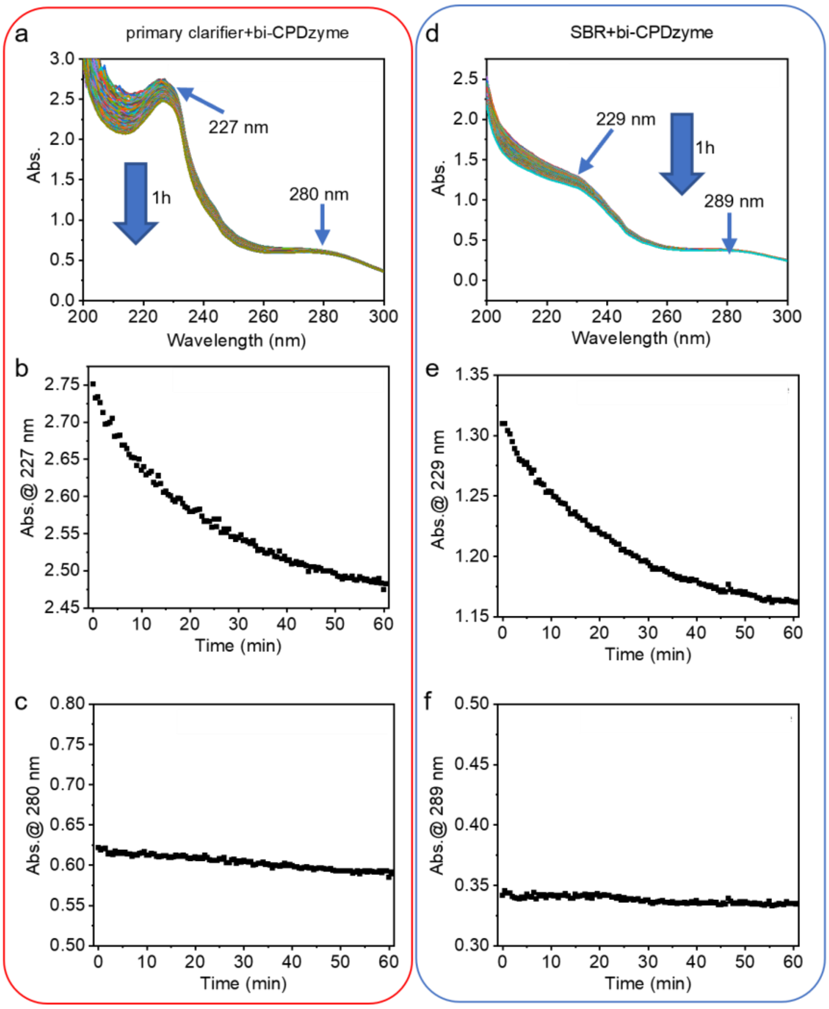

Figure S31. (a) The UV absorption spectra of primary clarifier wastewater treated with bi-CPDzyme *versus* time. Absorption values at (b) 227 nm and (c) 280 nm as function of time. (d) The UV absorption spectra of SBR wastewater treated with bi-CPDzyme *versus* time. Absorption values at (e) 229 nm and (f) 289 nm as a function of time.

As displayed in **Figure S31**, two wastewaters were treated by bi-CPDzyme for one hour, the absorbance of Konjugierte substances was decreased, while nearly no change of benzene ring ones, suggesting bi-CPDzyme can degrade those pollutants with Konjugierte absorption.

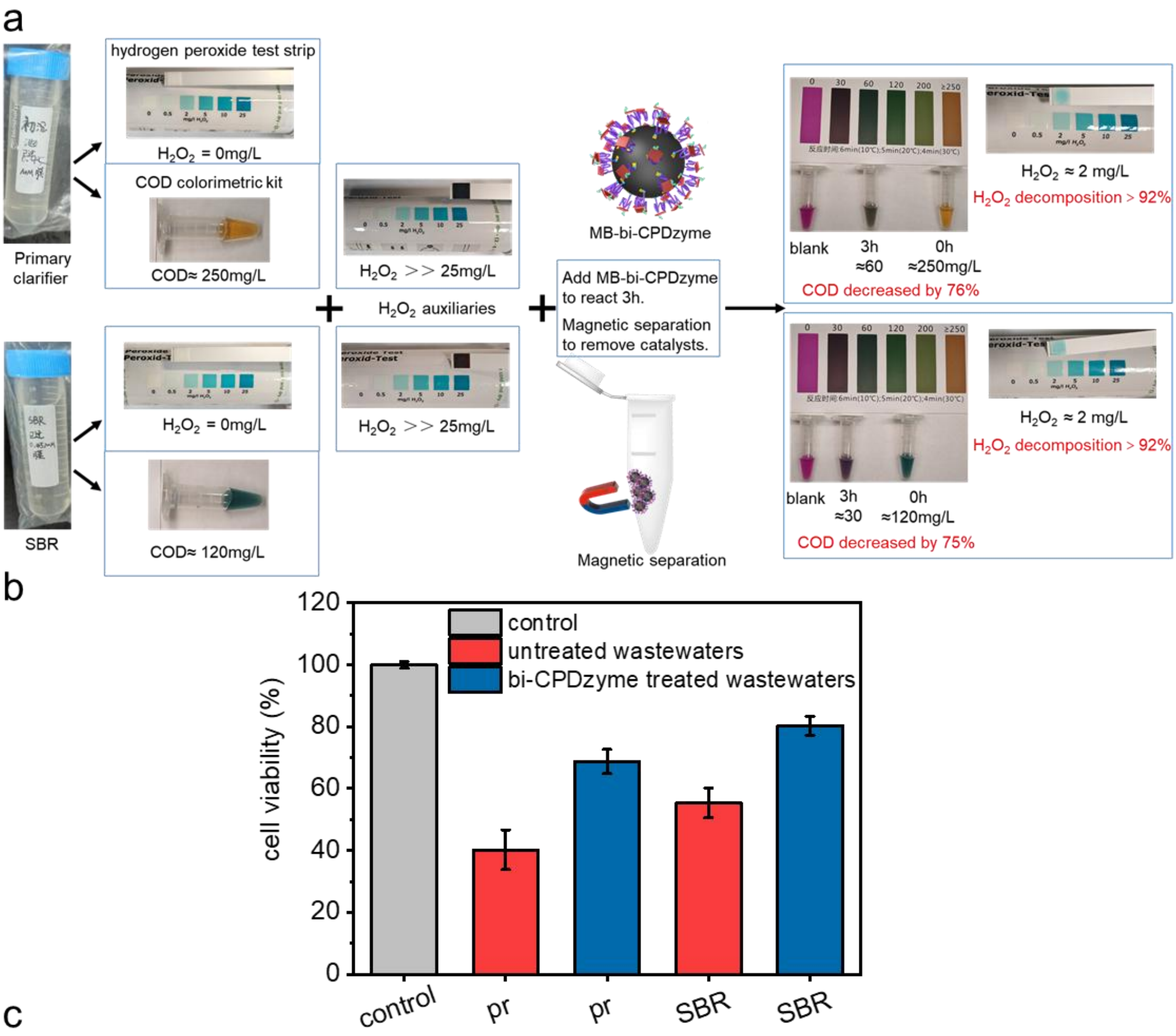

c

Cycle	1	2	3	4	5	6	7
H_2O_2 decomposition (%)	92	90	89	86	83	81	80
COD degradation (%)	76	75	73	70	69	68	67

Figure S32. (a) Initial H_2O_2 content and COD values of primary clarifier and SBR wastewaters, H_2O_2 content after addition of H_2O_2 auxiliaries, and changes in H_2O_2 content and COD values after treatment with bi-CPDzyme for three hours. (b) The cytotoxicity of primary clarifier and SBR wastewaters before and after 3 h treatment with bi-CPDzyme. (c) H_2O_2 decomposition and COD degradation rates by MB-bi-CPDzyme during consecutive catalytic cycles in primary clarifier wastewater.

Due to the long storage time of the actual samples, the H_2O_2 contained has self-decomposed. We artificially added H_2O_2 auxiliaries to investigate the bifunctional degradation ability of bi-CPDzyme in real samples. As shown in **Figure S32a**, the primary clarifier and SBR wastewaters had an initial value of H_2O_2 of 0 mg/L, and COD values of 250 and 120 mg/L, respectively. The H_2O_2 auxiliaries were added at this time, and the concentration of H_2O_2 were much greater than 25 mg/L. The wastewaters were treated with bi-CPDzymes for three hours, and then the catalysts were removed by magnetic separation. The

COD values after treatment were 60 and 30 mg/L, COD values decreased by 76% and 75%. The remaining amount of H_2O_2 was about 2 mg/L, and the decomposition rate of H_2O_2 was more than 92%. After 3 h of bi-CPDzyme treatment, the cell viability of primary clarifier and SBR wastewaters rose from 40.2 % and 55.3 % to 68.7 % and 80.3 %, respectively (**Figure S32b**).

Using primary clarifier wastewater as the treatment medium, the MB-bi-CPDzyme maintained 87% CAT activity (with H_2O_2 decomposition rate reaching 80%) and 88% POD activity (achieving 67% COD degradation efficiency) after seven consecutive catalytic cycles (**Figure S32c**), demonstrating the high efficiency, stability, and reusability of MB-bi-CPDzyme in continuous industrial operations.

Movie S1 (separate file). DFT calculations were employed to investigate the reaction mechanism between H_2O_2 and the G4-Hemin system, elucidating the formation of compound I.

Movie S2 (separate file). DFT calculations were conducted to explore the reaction pathway of H_2O_2 with the G4-Hemin-KHR system, with a focus on the formation of compound I.

Movie S3 (separate file). AIMD simulations were used to study the behavior and evolution of Fe-OH and OH^- within the G4-Hemin system, providing insights into the dynamic interactions.

Movie S4 (separate file). AIMD simulations were performed to investigate the process of proton removal from the Fe-OH group in the G4-Hemin-KHR system, revealing the underlying mechanisms of this deprotonation.

Table S1. The nucleic acid and amino acid sequences used in this work

Name	Sequences (5´–3´)(N-C)
**G4TC**	GGGTGGGTGGGTGGGTC
**G4-NH_2**	GGGTGGGTGGGTGGG-NH_2
**bio-T_{10}G4-NH_2**	bio-T_{10}GGGTGGGTGGGTGGG-NH_2
**bio-T_{10}PC-G4-NH_2**	bio-T_9/iPCLink/TGGGTGGGTGGGTGGG-NH_2 [a]
**KH**	Lys-His
**KR**	Lys-Arg
**KN**	Lys-Asn
**KS**	Lys-Ser
**KRH**	Lys-Arg-His
**KHR**	Lys-His-Arg
**KHS**	Lys-His-Ser
**KSH**	Lys-Ser-His
**KHN**	Lys-His-Asn
**KNH**	Lys-Asn-His
**KNRH**	Lys-Asn-Arg-His
**KNHR**	Lys-Asn-His-Arg
**KHRN**	Lys-His-Arg-Asn
**KHNR**	Lys-His-Asn-Arg
**KRHN**	Lys-Arg-His-Asn
**KRNH**	Lys-Arg-Asn-His
**KHNS**	Lys-His-Asn-Ser
**KHSN**	Lys-His-Ser-Asn
**KNHS**	Lys-Asn-His-Ser
**KNSH**	Lys-Asn-Ser-His
**KSHN**	Lys-Ser-His-Asn
**KSNH**	Lys-Ser-Asn-His
**KSHR**	Lys-Ser-His-Arg
**KSRH**	Lys-Ser-Arg-His
**KHSR**	Lys-His-Ser-Arg

**KRSH**	Lys-Arg-Ser-His
**KHRS**	Lys-His-Arg-Ser
**KRHS**	Lys-Arg-His-Ser
**KHRH**	Lys-His-Arg-His
**KRHR**	Lys-Arg-His-Arg
**KHNNH**	Lys-His-Asn-Asn-His
**KHSSH**	Lys-His-Ser-Ser-His
**KHRRH**	Lys-His-Arg-Arg-His
**KHRRH**[b]	Lys-His-Arg-Arg-His[b]
**HRKRH**	His-Arg-Lys-Arg-His
**RHKHR**	Arg-His-Lys-His-Arg
**HRRHK**	His-Arg-Arg-His-Lys
**KHRRHRH**	Lys-His-Arg-Arg-His-Arg-His
**KHRHRHR**	Lys-His-Arg-His-Arg-His-Arg
**HRRHKHR**	His-Arg-Arg-His-Lys-His-Arg
**RHKHRRH**	Arg-His-Lys-His-Arg-Arg-His
**HRRHKHRRH**	His-Arg-Arg-His-Lys-His-Arg-Arg-His
**HRRHKHRRHRH**	His-Arg-Arg-His-Lys-His-Arg-Arg-His-Arg-His
**HRHRRHKHRRH**	His-Arg-His-Arg-Arg-His-Lys-His-Arg-Arg-His

[a]*iPCLink represents photocleavage site.* [b]*D-type amino acids and all others are L-type amino acids.*

Table S2. Kinetic parameters of POD and CAT reactions catalyzed by different enzymes.

Catalyst	**POD activity**[a]			**CAT activity**[b]			Ref.
	K_m[c] (mM)	k_{cat} (s^{-1})	k_{cat}/K_m (s^{-1} mM^{-1})	K_m[c] (mM)	k_{cat} (s^{-1})	k_{cat}/K_m (s^{-1} mM^{-1})	
Natural HRP	0.11	521	4.7×10^{3}	-	-	-	1
Natural CAT	-	-	-	21	32891	1.57×10^{3}	This work
Natural KatG	0.06-1	7.7-25	13-417	3.1-4.7	2950-7770	$0.7\text{-}2.46\times10^{3}$	13
FeN_3P-SAzyme	443	5.28	1.2×10^{-2}	-	-	-	14
Co_3O_4 nanozyme	-	-	-	38.7	0.179	4.63×10^{-3}	15
Co-N_3PS SAzyme	-	-	-	6.1	520	85.2	
RuNC_Cl	1200	13.9	1.2×10^{-3}	-	-	-	16
FeNC	1916	7.14	3.7×10^{-3}	114.5	113.9	0.995	
FeN_4	12.45	0.25	2.0×10^{-2}	2.04	73.9	36.23	17
RhN_4	12.38	0.084	6.8×10^{-3}	1.33	535	402.3	
Cu-N-C	22.6	0.11	4.9×10^{-3}	183.9	1.1	5.98×10^{-3}	18
Cu-N/S-C	15.41	0.23	1.5×10^{-2}	13.32	5.19	0.39	
Mn_1/NHCS	48.16	0.12	2.5×10^{-3}	33.56	1.23	0.04	19
Mn_1-S/NSHCS	35.89	0.57	1.6×10^{-2}	23.69	10.19	0.43	
De novo enzyme	1.2	28.8	24	-	-	-	20,21
heme−peptide metalloenzyme	44.2	371	8.4	-	-	-	22
Biot2-FePP SavS112EK121H	0.4	5700	1.4×10^{4}	-	-	-	23
G4/Hemin	1.8-7.62	0.3-25.4	0.11-3.3	-	-	-	24
G4-Hemin	147	33.4	0.2	118	482	4.1	This work
bi-CPDzyme-	101	1245	12.3	104	6341	61	This work

[a]*Orange background are POD kinetic parameters.* [b]*Blue background are CAT kinetic parameters.* [c]*Substrates:H_2O_2 mM.*

Table S3. The catalytic cost performance of bi-CPDzyme.

bi-CPDzyme			
Manufacturer	Bide Pharmatech	Sangon Biotech	Genscript
Name	Hemin	G4-NH_2	HRRHKHRRH
Pack Size	25 g	100 nmol	1 g
Price (¥)	441	303.3	2545.38
Molecular Weight	651.94	/	1319.49
Unit Price (¥/g)	17.64	/	2545.38
Unit Price (¥/mol)	$1.15*10^4$	$3.03*10^9$	$3.36*10^6$
Unit Price of Catalyst (¥/mol $_{cat.}$)	$3.0334*10^9$		
k_{cat} (s^{-1})	1245 (POD), 6341(CAT)		
Price (¥/mol $_{sub.}$)	**$2.44*10^6$ (POD), $4.78*10^5$ (CAT)**		

Table S4. The catalytic cost performance of HRP and CAT.

	HRP (Peroxidase from horseradish)	CAT (Catalase from bovine liver)
Manufacturer	Sigma-Aldrich	Sigma-Aldrich
Name	P6782	C3155
Pack Size	5 mg	50 mg
Price (¥)	773.62	1790.99
Molecular Weight	44 kDa	250 kDa
Unit Price (¥/g)	$1.55*10^5$	$3.58*10^4$
Unit Price of Catalyst (¥/mol $_{cat.}$)	$6.81*10^9$	$8.95*10^9$
k_{cat} (s^{-1})	521	32891
Price (¥/mol $_{sub.}$)	**$1.31*10^7$**	**$2.72*10^5$**

Table S5. The catalytic cost performance of bi-CPDzyme and natural enzyme blend.

	bi-CDPzyme	HRP/ CAT blend	Cost percentage
Price (¥/mol $_{sub.}$)	**$2.918*10^{6}$ (POD+CAT)**	**$1.337*10^{7}$ (POD+CAT)**	**21.8%**

References

1. Zhang X, Qiu D, Chen J *et al.* Chimeric biocatalyst combining peptidic and nucleic acid components overcomes the performance and limitations of the native horseradish peroxidase. *J Am Chem Soc* 2023; **145**: 4517-26.

2. Kühne TD, Iannuzzi M, Del Ben M *et al.* CP2K: an electronic structure and molecular dynamics softwarepackage - quickstep: efficient and accurate electronic structure calculations. *J Chem Phys* 2020; **152**: 194103.

3. Humphrey W, Dalke A, Schulten K. VMD: visual molecular dynamics. *J Mol Graphics* 1996; **14:** 33−8.

4. Frisch MJ, Trucks GW, Schlegel HB *et al.* Gaussian 16A.03. wallingford, CT, 2016.

5. Lu T. A comprehensive electron wavefunction analysis toolbox for chemists, multiwfn. *J Chem Phys* 2024; **161:** 082503.

6. Zhang J, Lu T. Efficient evaluation of electrostatic potential with computerized optimized code. *Phys Chem Chem Phys* 2021; **23**: 20323-8.

7. Murray JS, Politzer P. The electrostatic potential: an overview. *Mol Sci* 2011; **1:** 153.

8. Eberhardt J, Santos-Martins D, Tillack A F *et al.* Autodock vina 1.2.0: new docking methods, expanded force field, and python bindings. *J Chem Inf Model* 2021; **61**: 3891−8.

9. Trott O, Olson A J. Autodock vina: improving the speed and accuracy of docking with a new scoring function, efficient optimization, and multithreading. *J Comput Chem* 2010; **31**: 455–61.

10. Gao L, Zhuang J, Nie L *et al.* Intrinsic peroxidase-like activity of ferromagnetic nanoparticles. *Nat Nanotechnol* 2007; **2**: 577−83.

11. Qiu Y, Yuan B, Mi H *et al.* An atomic insight into the confusion on the activity of Fe_3O_4 nanoparticles as peroxidase mimetics and their comparison with horseradish peroxidase. *J Phys Chem Lett* 2022; **13**: 8872−8.

12. Jia Y, Shan C, Fu W *et al.* Occurrences and fates of per- and polyfluoralkyl substances in textile dyeing wastewater along full-scale treatment processes. *Water Res* 2023; **242**: 120289.

13. Singh R, Wiseman B, Deemagarn T *et al.* Comparative study of catalase-peroxidases (KatGs). *Arch Biochem Biophys* 2008; **471**: 207-14.

14. Ji S, Jiang B, Hao H *et al.* Matching the kinetics of natural enzymes with a single-atom iron nanozyme. *Nat Catal* 2021; **4**: 407-17.

15. Chen Y, Jiang B, Hao H *et al.* Atomic-level regulation of cobalt single-atom nanozymes: engineering high-efficiency catalase mimics. *Angew Chem Int Ed* 2023; **62**: 202301879.

16. Park S, Shim K, Nguyen P *et al.* Breaking the selectivity barrier of single-atom nanozymes through out-of-plane ligand coordination. *Adv Mater* 2025; **37**: 2506480.

17. Zhang S, Li Y, Sun S *et al.* Single-atom nnanozymes catalytically surpassing naturally occurring enzymes as sustained stitching for brain trauma. *Nat Commun* 2022; **13**: 4744.

18. Kim K, Lee J, Park O *et al.* Geometric tuning of single-atom FeN4 sites via edge-generation enhances multi-enzymatic properties. *Adv Mater* 2023; **35**: 2207666.

19. Zhang Z, Li F, Xi S *et al.* Atomic metal–nonmetal catalytic pair cooperatively drives efficient enzyme-mimetic catalysis. *Angew Chem Int Ed* 2025; **64**: 202508651.

20. Watkins DW, Jenkins JMX, Grayson KJ *et al.* Construction and in vivo assembly of a catalytically proficient and hyperthermostable de novo enzyme. *Nat Commun* 2017; **8**: 358.

21. Hindson SA, Bunzel HA, Frank B *et al.* Rigidifying a de novo enzyme increases activity and induces a negative activation heat capacity. *ACS Catal* 2021; **11**: 11532−41.

22. Nastri F, Lista L, Ringhieri P *et al.* A heme−peptide metalloenzyme mimetic with natural peroxidase like activity. *Chem Eur J* 2011; **17**: 4444−53.

23. Mukherjee M, Waser V, Morris E *et al.* Artificial peroxidase based on the biotin−streptavidin technology that rivals the efficiency of natural peroxidases. *ACS Catal* 2024; **14**: 16266−76.

24. Chen J, Wang J, Van der Lubbe SCC *et al.* A push- pull mechanism helps design highly competent g-quadruplex-DNA catalysts. *CCS Chem* 2021; **3**: 2183−93.
